# Perinatal health outcomes and care among asylum seekers and refugees: a systematic review of systematic reviews

**DOI:** 10.1186/s12916-018-1064-0

**Published:** 2018-06-12

**Authors:** Nicola Heslehurst, Heather Brown, Augustina Pemu, Hayley Coleman, Judith Rankin

**Affiliations:** Institute of Health & Society, Newcastle University, Baddiley-Clark Building, Richardson Road, Newcastle upon Tyne, NE2 4AX UK

**Keywords:** Migrant health, Asylum seeker, Refugee, Perinatal health, Pregnancy, Access to care, Systematic review

## Abstract

**Background:**

Global migration is at an all-time high with implications for perinatal health. Migrant women, especially asylum seekers and refugees, represent a particularly vulnerable group. Understanding the impact on the perinatal health of women and offspring is an important prerequisite to improving care and outcomes. The aim of this systematic review was to summarise the current evidence base on perinatal health outcomes and care among women with asylum seeker or refugee status.

**Methods:**

Twelve electronic database, reference list and citation searches (1 January 2007–July 2017) were carried out between June and July 2017. Quantitative and qualitative systematic reviews, published in the English language, were included if they reported perinatal health outcomes or care and clearly stated that they included asylum seekers or refugees. Screening for eligibility, data extraction, quality appraisal and evidence synthesis were carried out in duplicate. The results were summarised narratively.

**Results:**

Among 3415 records screened, 29 systematic reviews met the inclusion criteria. Only one exclusively focussed on asylum seekers; the remaining reviews grouped asylum seekers and refugees with wider migrant populations. Perinatal outcomes were predominantly worse among migrant women, particularly mental health, maternal mortality, preterm birth and congenital anomalies. Access and use of care was obstructed by structural, organisational, social, personal and cultural barriers. Migrant women’s experiences of care included negative communication, discrimination, poor relationships with health professionals, cultural clashes and negative experiences of clinical intervention. Additional data for asylum seekers and refugees demonstrated complex obstetric issues, sexual assault, offspring mortality, unwanted pregnancy, poverty, social isolation and experiences of racism, prejudice and stereotyping within perinatal healthcare.

**Conclusions:**

This review identified adverse pregnancy outcomes among asylum seeker and refugee women, representing a double burden of inequality for one of the most globally vulnerable groups of women. Improvements in the provision of perinatal healthcare could reduce inequalities in adverse outcomes and improve women’s experiences of care. Strategies to overcome barriers to accessing care require immediate attention. The systematic review evidence base is limited by combining heterogeneous migrant, asylum seeker and refugee populations, inconsistent use of definitions and limited data on some perinatal outcomes and risk factors. Future research needs to overcome these limitations to improve data quality and address inequalities.

**Systematic registration:**

Systematic review registration number: PROSPERO CRD42017073315.

**Electronic supplementary material:**

The online version of this article (10.1186/s12916-018-1064-0) contains supplementary material, which is available to authorized users.

## Background

Gobalisation, poor living conditions, war and conflict are major factors contributing to forced migration. In 2016, the number of people displaced by conflict and persecution worldwide was estimated to be 65.6 million. Of these 2.8 million were estimated to be asylum seekers and 22.5 million refugees, which the United Nations High Commissioner for Refugees (UNHCR) suggests is the highest level ever recorded [1]. Among this population, 49% of refugees were women, a similar proportion as reported annually since 2003 [1]. The impact of migration on health is far-reaching, making migrant populations particularly vulnerable, fuelling health inequalities and resulting in serious implications for global health.

Research on migrant populations is challenged by the diverse terminology and definitions used. For the purposes of this systematic review, we use the following UNHCR definitions [1]:

- *Asylum seekers* are individuals who have sought international protection and whose claims for refugee status have not yet been determined, irrespective of when they may have been lodged. An *asylum seeker* has applied for asylum on the grounds of persecution in their home country relating to their race, religion, nationality, political belief or membership of a particular social group. This population remains classified as asylum seeker for as long as the application is pending.
- *Refugees* have been forced to leave their country in order to escape war, persecution or natural disaster. The 1951 Convention relating to the Status of Refugees describes a refugee as “a person who owing to a well-founded fear of being persecuted for reasons of race, religion, nationality, membership of a particular social group, or political opinion, is outside the country of this nationality and is unable to or, owing to such fear, is unwilling to avail himself of the protection of that country”. A *refugee* is an asylum seeker whose application has been successful.
- *Migrants* include those who move, either temporarily or permanently from one place, area or country of residence to another for reasons such as work or seeking a better life (i.e. economic migrants), for family reasons or to study. People also migrate to flee conflict or persecution, which is where the definition converges with the terms refugee and asylum seeker.

Timely access to perinatal healthcare is an effective method to optimise pregnancy outcomes and the lifelong health of women and their offspring. Late access to maternity care can result in adverse perinatal outcomes. Vulnerable pregnant women, including women with asylum seeker and refugee status, face barriers to accessing healthcare [2] including maternity care [3]. A recent report of vulnerable women in social crisis in Europe included pregnant women seeking or having been refused asylum and found that 65% had no access to antenatal care, 42% accessed care after 12 weeks of pregnancy and two thirds were classified as being 'at risk' requiring urgent or semi-urgent care [4]. This disparity in access to, and use of, perinatal healthcare can lead to significant health inequalities. Failure to effectively reach and provide optimal perinatal care for women with asylum seeker and refugee status will result in failure to reduce health inequalities for this vulnerable group of women and their babies.

There has been a recent escalation of systematic reviews investigating different aspects of perinatal health in women who have migrated, which includes asylum seeker and refugee populations. For example, multiple systematic reviews were published in 2016 and 2017 on topics including perinatal health outcomes [5–8] and experiences of antenatal care [9–11]. However, there is a lack of published systematic reviews that explicitly address pregnancy among asylum seeker and refugee populations, and there is a tendency to group all migrant populations together in syntheses. Given this, we have chosen to undertake a systematic review of systematic reviews to assess the research gaps and provide direction to future research specifically relating to women with asylum seeker and refugee status. The aim of this systematic review was to provide an overview of the existing evidence base drawn from systematic reviews that have examined perinatal healthcare and outcomes among women with asylum seeker or refugee status.

## Methods

The Joanna Briggs Institute (JBI) methodology for umbrella reviews was used to guide this systematic review of systematic reviews [12]. The Preferred Reporting Items for Systematic Reviews and Meta-Analyses (PRISMA) reporting guidelines and checklist (Additional file 1) have been used to report each stage of the systematic review methods and findings [13]. The protocol for this systematic review has been registered in the PROSPERO database (CRD42017073315).

### Identification of studies

Electronic bibliographic databases were searched using PICOS criteria: Population (asylum seekers or refugees); Intervention (pregnancy); Comparator (non-asylum seekers or refugees for quantitative reviews only, no comparator group required for qualitative reviews); Outcome (defined as selected perinatal health outcomes or care); Study design (quantitative, qualitative or mixed methods systematic reviews). A search strategy for database-specific search terms and subject headings was developed with the support of an information scientist for the databases MEDLINE, Embase, Scopus, Cumulative Index to Nursing and Allied Health Literature, JBI database, PROSPERO, Cochrane Database of Systematic Reviews, Google Scholar, Science Direct, Web of Science, PubMed and ProQuest (see Additional file 2 for database search terms).

Database searches were supplemented with hand searching of the reference lists of all included systematic reviews to identify any further relevant reviews. All included systematic reviews were also subjected to citation searches using all citations produced by Google Scholar. Any systematic reviews identified by the supplementary searches which met the inclusion criteria were also subject to reference list and citation searches until no further eligible reviews were identified. The detailed search strategy was carried out between June and July 2017 and restricted to systematic reviews published within the past 10 years (since January 2007) as per the JBI recommendation [12]. No restrictions were placed on country or region of study or on low-, middle- or high-income status of the host countries. Inclusion criteria were as follows:

- Systematic reviews with a quantitative, qualitative or mixed methods evidence synthesis
- Published in the English language
- Included any perinatal health outcomes (e.g. postnatal depression, low birth weight) or perinatal care (e.g. access to maternity services, experiences of care) during the preconception, antenatal and postnatal periods
- Clearly stated that women with asylum seeker or refugee status were populations within the included studies. This included reviews of migrant women where asylum seekers and refugees were part of the included population

Reviews were excluded if they were:

- Scoping reviews which aimed to identify the extent and nature of the evidence base without a formal evidence synthesis
- Published abstracts without full texts and protocols of systematic reviews. We searched for any subsequent full text publications of these works
- Reviews that focussed on refugees living in camps

**Fig. 1:**
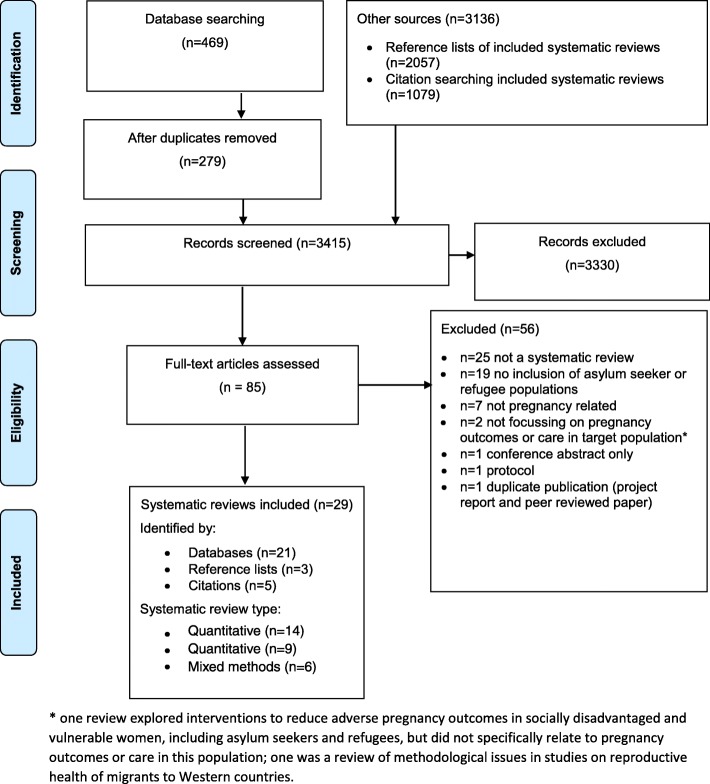
PRISMA flowchart of searches, screening, and inclusion and exclusion of studies

Two authors independently screened titles, abstracts and full texts for inclusion in the review. Disagreements regarding eligibility for inclusion were resolved through discussion; a third independent reviewer was available where no agreement could be reached (not required). References were managed and recorded in EndNote version X7. The flow of reviews through each stage of the searches and screening and the reasons for exclusions are presented using a PRISMA diagram (Fig. 1). Data extraction and quality assessments were carried out in duplicate for all included systematic reviews. Independent data extractions and quality assessments were combined by two authors and agreed with recourse to a third reviewer if no agreement could be reached (not required).

### Quality assessment

The JBI Critical Appraisal Checklist for Systematic Reviews and Research Syntheses [12] was used for quality assessment. The checklist comprises 11 questions relating to methodological rigor, transparency of reporting and appropriateness of conclusions and recommendations, with options of ’yes’ if the review clearly meets the checklist criteria and ’no’, ’unclear’ or ’not applicable’ if the review does not clearly meet the criteria (see Additional file 3). The reviews were awarded a score of 1 for each checklist criterion clearly met, with a maximum possible score of 11. The reviews were considered to be of high quality if they scored 8–11, moderate quality for scores of 4–7 and low quality for scores of 0–3. No reviews were excluded based on quality score. The percentage of included reviews meeting the criteria was calculated for each of the 11 checklist questions.

### Data extraction

The JBI umbrella review data extraction form was adapted to meet the needs of this mixed methods systematic review of systematic reviews (see Additional file 3). The following data were extracted for each included systematic review: aim, objectives and focus of the review including review type, aims, objectives, type/definition of included population, inclusion and exclusion criteria and outcomes included in search strategy; search details including date range of the search, search strategy and restrictions to the search; appraisal rating including whether the quality appraisal was reported, what method/tool was used and summary of quality of included studies; key results including the number of included studies, publication date range, sample size, host countries, description of included population, summary results and conclusions for the overall population and also detailed results and conclusions explicitly relating to asylum seekers and refugee populations.

We implemented a process of systematically extracting data which was directly relevant to women with asylum seeker and refugee status for all of the systematic reviews which combined data from multiple populations in their syntheses (e.g. migrants including asylum seekers and refugees). This involved two stages of searching for relevant data in the tables, figures and narrative in the results, discussion and conclusions sections of the included systematic reviews. First, the relevant sections of the reviews were searched for data that the authors had explicitly described as being relevant to asylum seeker or refugee populations, and these data were extracted. The second stage involved identifying whether any of the included studies in the systematic reviews were exclusively among asylum seeker or refugee populations. When studies which were exclusively among these populations were identified, data were extracted for any results which had cited these studies as part of the evidence base which informed that specific result. This second stage was only carried out if we were confident that the population of the included study were exclusively asylum seekers and/or refugees; for example, data were not extracted for studies which included migrants and refugees, as we could not be confident that the data that had informed the result originated from women who were migrants or refugees.

### Evidence synthesis

Evidence synthesis in systematic reviews of systematic reviews should provide a summary of existing research syntheses in tabular format with a more detailed narrative description of the systematic review characteristics and relevant quantitative and qualitative results [12]. The results have therefore been summarised in tables to describe the characteristics of the included systematic reviews, results for overall populations included in the systematic reviews and also results explicitly relevant to women with asylum seeker and refugee status. Tables are supplemented with a narrative discussion of the included systematic reviews grouped by the review themes of perinatal health outcomes and perinatal healthcare access and experiences for women who are migrants and for asylum seekers and refugees. Each theme has several data-driven sub-themes. As per reporting recommendations [12], any overlap in original research studies in the included systematic reviews is reported in the results table and narrative for the asylum seeker- and refugee-specific data. Evidence synthesis was carried out in duplicate. The first stage of synthesis was performed by JR, HB and AP to group the systematic review data into the review themes and to provide a descriptive summary of the topic-specific data. The second stage involved NH validating the review themes and performing a detailed synthesis of sub-theme data reported in the systematic reviews.

## Results

### Included systematic reviews

A total of 3415 records were identified by searches, of which 29 were systematic reviews which met the inclusion criteria (Fig. 1). Twenty-one systematic reviews were identified through the database searches, three from reference list and five from citation searching. Fourteen of the included reviews were quantitative, eight of which included a meta-analysis, nine were qualitative and six involved mixed methods (Table 1). The systematic reviews were published between 2009 and 2017; the fewest reviews were published in 2011 and 2012 (*n* = 1 per year), and most were published in 2017 (*n* = 7 published between January and the date of the searches in July and August). The number of studies included in the reviews ranged from eight to 133, and the publication years of the included studies were from 1956 to 2016. Only one systematic review identified was exclusively focussed on women with asylum seeker status [14]. The populations included in the remaining systematic reviews were migrant populations including women with asylum seeker and/or refugee status (*n* = 27) and marginalised women (*n* = 1), which included those with asylum seeker and/or refugee status along with women who had experienced domestic violence, minority ethnic groups, travelling communities, women of low income, those with substance abuse problems, teenagers and women who were homeless. There was no consistent definition in the use of terminology to describe women, and many reviews did not adequately define their populations. We use the terms asylum seeker, refugee and migrant as previously defined, and the term ’women in the host country’ to collectively describe comparison groups that have been reported in the systematic reviews which include a range of definitions including non-migrant, native-born, etc. (see Additional file 4 for a detailed description of the study populations).

**Table 1:** Summary of included systematic reviews

Author, year	Aim of the systematic review	Methods	Population included	Search strategy (years, databases and supplementary searches)
Alhasanat and Fry-McComish 2015 [16]	To identify the prevalence and risk factors for postnatal depression amongst immigrant women in industrialised countries and compare it with prevalence and risk factors amongst Arab women in their home countries	Quantitative with narrative	Migrant including asylum seekers and/or refugees	1990–2013 Four databases searched No supplementary searches
Anderson et al. 2017 [5]	To evaluate the prevalence and risk factors of mental disorders in the perinatal period amongst migrant women	Quantitative with meta-analysis	Migrant including asylum seekers and/or refugees	From inception of database to Oct 2015 Six databases searched No supplementary searches
Aubrey et al. 2017 [9]	To broadly explore and synthesise current evidence surrounding women’s preference for female physicians in obstetrics and gynaecology	Mixed methods	Migrant including asylum seekers and/or refugees	From inception of database Five databases searched Supplementary searches: reference list, citations
Balaam et al. 2013 [30]	To explore migrant women’s perceptions of their needs and experiences related to pregnancy and childbirth	Qualitative	Migrant including asylum seekers and/or refugees	1996–2010 Seven databases searched No supplementary searches
Bollini et al. 2009 [26]	To explore whether differences in pregnancy outcomes observed across receiving countries in Europe are associated with varying degrees of implementation of integration policies	Quantitative with meta-analysis	Migrant including asylum seekers and/or refugees	1966–2004 One database searched No supplementary searches
Collins et al. 2011 [18]	To review the rates and risk factors associated with postnatal depression in refugees, asylum seekers and migrant women	Quantitative with narrative	Migrant including asylum seekers and/or refugees	1990–2009 Ten databases searched No supplementary searches
De Maio 2010 [19]	This review investigates the health of immigrants to Canada by critically examining differences in health status between immigrants and the native-born population and by tracing how the health of immigrants changes after settling in the country	Quantitative with narrative	Migrant including asylum seekers and/or refugees	1990–2010 Four databases searched Supplementary searches: reference list
Downe et al. 2009 [31]	To locate and synthesise qualitative accounts of barriers to antenatal care as reported by high-risk, marginalised, pregnant women in the UK	Qualitative	Marginalised populations including asylum seekers and/or refugees	1980–2007 Seven databases searched Supplementary searches: reference list
Falah-Hassani et al. 2015 [17]	Estimate the prevalence of postpartum depressive systems in immigrant women. Compare this prevalence to non-immigrant women. Determine risk factors for postpartum depressive systems in immigrant women	Quantitative with meta-analysis	Migrant including asylum seekers and/or refugees	1950–2014 Seven databases searched Supplementary searches: reference list
Fellmeth et al. 2017 [6]	Summarise and synthesise evidence on prevalence, associated factors and effectiveness of interventions for any perinatal mental disorder in migrant women	Quantitative with meta-analysis	Migrant including asylum seekers and/or refugees	No specific start date until January 2015 Eight databases searched Supplementary searches: hand searching journals, reference list
Gagnon et al. 2009 [25]	To assess whether migrants in western industrialised countries have consistently poorer perinatal health than receiving-country women	Quantitative with meta-analysis	Migrant including asylum seekers and/or refugees	1995–2008 Four databases searched Supplementary searches: reference list, citations
Gissler et al. 2009 [27]	To determine if migrants in western industrialised countries have consistently higher risks of stillbirth, neonatal mortality or infant mortality; if there are migrant sub-groups at potentially higher risk; and what might be the explanations for any risk differences found	Quantitative with meta-analysis	Migrant including asylum seekers and/or refugees	1995–2006 Four databases searched Supplementary searches: reference list
Hadgkiss and Renzaho 2014 [14]	To document physical health problems that asylum seekers experience on settlement in the community and to assess their utilisation of healthcare services and barriers to care, in an international context	Mixed methods	Asylum seekers	2002–2012 Four databases searched Supplementary searches: reference list
Heaman et al. 2013 [35]	Do migrant women in Western industrialised countries have higher odds of inadequate prenatal care compared to receiving-country women, and what factors are associated with inadequate prenatal care amongst migrant women in Western industrialised countries?	Quantitative with narrative	Migrant including asylum seekers and/or refugees	1995–2010 Three databases searched Supplementary searches: reference list
Higginbottom et al. 2015 [36]	What are the experiences of immigrant women in Canada in accessing and navigating maternity and healthcare services from conception to 6 months postpartum?	Mixed methods	Migrant including asylum seekers and/or refugees	Inception to 2013 Ten databases searched Supplementary searches: hand searches within journal websites
Higginbottom et al. 2014 [32]	To synthesise qualitative literature to describe how immigrant women experience maternity services in Canada	Qualitative	Migrant including asylum seekers and/or refugees	Inception to March 2012 Eight databases searched Supplementary searches: contacting authors, reference list
Higginbottom et al. 2012 [23]	To identify and descriptively synthesise current empirical literature on immigrants’ experiences of maternity healthcare services in Canada, to outline practice implications and/or to offer recommendations for future research	Mixed methods	Migrant including asylum seekers and/or refugees	2000–2010 Five databases searched Supplementary searches: reference list, citations
Mengesha et al. 2016 [10]	To identify studies that focussed on the views and experiences of culturally and linguistically diverse women in accessing sexual and reproductive health care in Australia	Mixed methods	Migrant including asylum seekers and/or refugees	April 1990–May 2015 Seven databases searched Supplementary searches: reference list
Merry et al. 2013 [28]	To determine if migrants in Western industrialised countries consistently have different rates of caesarean than receiving-country-born women and to identify the reasons that explain these differences	Quantitative with meta-analysis	Migrant including asylum seekers and/or refugees	Inception to Jan 2012 Eleven databases searched Supplementary searches: reference list
Merry et al. 2016 [7]	To provide a synthesis to what is known regarding caesarean births amongst migrants living in high-income countries	Quantitative with narrative	Migrant including asylum seekers and/or refugees	2012–2015 Thirteen databases searched No supplementary searches
Nilaweera et al. 2014 [22]	To summarise the available evidence about the prevalence, nature and determinants of postpartum mental health problems amongst women born in South Asian countries who had migrated to high-income countries, and identify barriers and enablers to seeking health care for these difficulties	Mixed methods	Migrant including asylum seekers and/or refugees	Inception to February 2013 Four databases searched No supplementary searches
Pedersen et al. 2014 [24]	A meta-analysis of all published observational studies from Western European countries comparing the risk of maternal mortality between the receiving-country women and a defined migrant population	Quantitative with meta-analysis	Migrant including asylum seekers and/or refugees	1970–2013 Four databases searched Supplementary searches: reference list
Schmied et al. 2017 [8]	To report the findings of a meta-ethnographic study of the experiences, meanings and ways of ‘dealing with’ symptoms or a diagnosis of postnatal depression amongst migrant women living in high-income countries with a view to informing culturally appropriate health service design and delivery	Qualitative	Migrant including asylum seekers and/or refugees	1999–2016 Five databases searched Supplementary searches: reference list, citations
Small et al. 2014 [33]	There were two review questions: 1. What do immigrant and non-immigrant women want from their maternity care? 2. How do immigrant and non-immigrant women’s experiences and ratings of care compare, both within and across included countries?	Qualitative	Migrant including asylum seekers and/or refugees	1989–2011 Five databases searched No supplementary searches
Tobin et al. 2017 [20]	To synthesise qualitative research on refugee and immigrant women’s experiences of postpartum depression to gain insight into the unique needs of this group of women	Qualitative	Migrant including asylum seekers and/or refugees	2004–2014 Five databases searched Supplementary searches: reference list
Villalonga-Olives et al. 2016 [29]	To discuss differences between the USA and Europe regarding reproductive health outcomes of immigrants and to elucidate why these differences occur	Quantitative with narrative	Migrant including asylum seekers and/or refugees	Dates not clear Two databases searched No supplementary searches
Wikberg and Bondas 2010 [34]	To explore and describe a patient perspective in research on intercultural caring in maternity care	Qualitative	Migrant including asylum seekers and/or refugees	1995–2009 Twelve databases searched Supplementary searches: reference list
Winn et al. 2017 [11]	To understand the experiences of pregnant immigrant women accessing perinatal care in North America	Qualitative	Migrant including asylum seekers and/or refugees	Inception to July 2016 Five databases searched Supplementary searches: reference list
Wittkowski et al. 2017 [21]	To appraise and assimilate qualitative findings of postnatal depression in immigrant mothers	Qualitative	Migrant including asylum seekers and/or refugees	1990–2014 Six databases searched Supplementary searches: reference list

### Quality of evidence

The quality scores ranged from six to 11 (*n* = 10 categorised as moderate quality, *n* = 19 high quality, Additional file 5). Of the 11 questions in the JBI Critical Appraisal Checklist for Systematic Reviews and Research Syntheses, all of the included reviews scored ‘yes’ in four questions (is the review question clearly stated; were the methods used to combine the studies appropriate; were recommendations for policy and/or practice supported by the reported data; and were the specific directives for new research appropriate?), while only six reviews scored ‘yes’ for the question ’was the likelihood of publication bias assessed?’ (see Additional file 5). Additionally, only 14 reviews used methods to minimise data extraction errors (e.g. duplicate data extraction), 18 carried out quality appraisal and only 21 used adequate sources and resources to search for studies (e.g. database searches supplemented with additional search methods) as recommended in guidelines for systematic reviews of observational studies [15].

### Perinatal health outcomes amongst women who are migrants (including asylum seekers and refugees)

Nineteen systematic reviews reported perinatal health outcomes including perinatal mental health, mortality (maternal and offspring), mode of delivery, birth weight, preterm birth, congenital anomalies and additional morbidities. The results are summarised in Table 2, and a narrative summary is presented for each outcome.

**Table 2:** Summary of results for all included study populations (migrant including asylum seeker and refugee women)

Author, year	Number of studies	Publication date range	Sample size^1^	Topic area of results	Summary of author conclusions
Alhasanat and Fry-McComish 2015 [16]	26	1998–2013 (date range of migrant studies)	9089	Perinatal health outcomes (mental health); access, utilisation and experience of perinatal healthcare	Some similarities in the risk factors for postnatal depression amongst migrant women and Arabic women in their country of birth: lack of social support, stressful life events, lack of emotional support from the partner, history of antenatal depression and marital dissatisfaction. Immigration stress and lack of access to health care services were found amongst migrant women. Lack of social support was more predominant in studies on migrant women
Anderson et al. 2017 [5]	53	1986–2015	119,076 (for the 52 studies which reported sample size)	Perinatal health outcomes (mental health)	Depression is common amongst pregnant and postpartum migrant women, although there is no evidence for an overall increased risk of depression amongst migrant women when compared to non-migrant women
Aubrey et al. 2017 [9]	54	2002–2016 (data for only 10 included studies reported)	Not reported	Access to and utilisation of perinatal healthcare	A key finding of both qualitative and quantitative studies was a preference for female providers because of religious reasons and comfort with a female provider. Provider competence was prioritised over gender
Balaam et al. 2013 [30]	16	2000–2010	393 (excluding men and health professionals)	Access, utilisation and experience of perinatal healthcare	Migrant women’s vulnerable situation when pregnant and giving birth must be improved
Bollini et al. 2009 [26]	65	1966–2004	18,322,978 women including 1,632,401 migrant women	Perinatal health outcomes (neonatal intensive care, offspring mortality, preterm birth, low birth weight, congenital anomalies, postpartum haemorrhage)	Risk ratios for low birth weight, preterm delivery, perinatal mortality and congenital anomalies between immigrant and native-born women were more similar in countries with strong integration policies. There was a migrant penalty for those European countries with weak integration policies
Collins et al. 2011 [18]	8	1998–2008	4574 (for the 7 studies which reported sample size)	Perinatal health outcomes (mental health)	Nearly all studies found rates of probable postnatal depression were higher in migrant women than native-born women
De Maio 2010 [19]	51	2006–2010	Not reported	Perinatal health outcomes (mental health, low birth weight, preterm birth, placental dysfunction); access to and utilisation of perinatal healthcare	Mental health issues are less prevalent amongst migrants than the Canadian-born population. However, this advantage diminishes as length of residence in Canada increases. Living in areas with a high density of migrants may help immigrants to retain this advantage
Downe et al. 2009 [31]	8	1998–2006	569 (excluding men and health professionals)	Perinatal healthcare access and experiences	A non-threatening, non-judgemental antenatal service run by culturally sensitive staff may increase access to antenatal care for marginalised women. Multiagency initiatives aimed at raising awareness of, and providing access to, antenatal care may also increase uptake
Falah-Hassani et al. 2015 [17]	24	1995–2013	63,926	Perinatal health outcomes (mental health)	The prevalence of depressive symptoms is 1.5–2.0 higher in migrant women compared with non-migrant women. Migrant women were more likely to develop depressive symptoms if they had shorter residency in the destination country, lower levels of social support, poorer marital adjustment and insufficient household income
Fellmeth et al. 2017 [6]	45	1986–2013	19,439 (including 7985 migrant)	Perinatal health outcomes (mental health)	Higher prevalence of postnatal depression in migrant women. Local language ability, length of residency and adhering to traditional birth practices were protective factors
Gagnon et al. 2009 [25]	133	1968–2005	20,152,134	Perinatal health outcomes (maternal and offspring mortality, mode of delivery, low birth weight, preterm birth, maternal health, congenital anomalies, maternal and infant infections, infant morbidities); access to and utilisation of perinatal healthcare	Of 9 outcome categories, 2 appear to be better amongst migrant women (health-promoting behaviour and birth weight), 6 appear worse (infection, congenital anomalies and infant morbidity, prenatal care, maternal health, feto-infant mortality and mode of delivery) and 1 did not differ in most studies (preterm birth)
Gissler et al. 2009 [27]	34	1980–2002	Not reported	Perinatal health outcomes (offspring mortality)	In the European studies, all non-refugee migrants had higher crude stillbirth rates, perinatal mortality rates, neonatal mortality rates and infant mortality rates
Hadgkiss and Renzaho 2014 [14]	32	2002–2012	Not reported	Perinatal health outcomes (offspring mortality, mode of delivery, birth weight, preterm birth, complex obstetric issues)	This study highlights the health inequities faced by asylum seekers residing in the communities of host countries, internationally
Heaman et al. 2013 [35]	29	1996–2007	24,362,611	Access to and utilisation of perinatal healthcare	Migrant women were more likely to receive inadequate prenatal care than receiving-country women. Inadequate prenatal care varied widely by country of birth, indicating that this is not a homogeneous group
Higginbottom, et al. 2012 [23]	30	Not reported	Not reported	Perinatal health outcomes (mental health); access to and utilisation of perinatal healthcare	New migrants are ten times more likely than Canadian-born women to experience personal barriers when accessing healthcare. Language is a particular problem, and current interpreting services are either underutilised or unavailable
Higginbottom et al. 2014 [32]	22	1990–2011	510 (for 21 studies that reported data, excluding 2 studies exclusively with health professionals)	Access, utilisation and experience of perinatal healthcare	Experiences in maternity healthcare for migrant women are deeply embedded in the social position of the women which influences the availability of social supports, communication possibilities with health professionals and socio-economic status, all of which relate to the organisational environment. Furthermore, migrants and healthcare staff have different beliefs and values which form their perceptions on how maternity healthcare should be provided. Cultural knowledge, beliefs, religious and traditional customs were most relevant for migrants, whereas healthcare staff emphasise biomedical needs
Higginbottom et al. 2015 [36]	24	1995–2011	10,339	Access, utilisation and experience of perinatal healthcare	Analysis of these 24 studies led to the development of five interrelated themes: utilisation of prenatal care and educational classes; adequacy of perinatal care; barriers to maternity care in the pre- and postnatal periods; isolation and limited social support; and outcomes related to the access to and the use of services
Mengesha et al. 2016 [10]	22	1998–2014	1943	Access, utilisation and experience of perinatal healthcare	Although culturally and linguistically diverse women in Australia have the opportunity to obtain necessary health services, they experience numerous barriers in accessing and utilising sexual and reproductive healthcare
Merry et al. 2013 [28]	76	1956–2010	1,029,454	Perinatal health outcomes (mode of delivery)	Sub-Saharan African, Somali and South Asian migrants consistently have higher caesarean rates while Eastern-European and Vietnamese migrants have lower overall caesarean rates compared to receiving-country-born women. North African, West Asian and Latin American migrant women have higher emergency caesarean rates
Merry et al. 2016 [7]	33	2012–2015	Not reported	Perinatal health outcomes (mode of delivery)	Women from sub-Saharan Africa and South Asia consistently show overall higher rates of caesarean compared with non-migrant women. Women from Latin America, North Africa and Middle East consistently show higher rates of emergency caesarean. Higher rates are more common with emergency caesareans than with planned caesareans
Nilaweera et al. 2014 [22]	15	2003–2012	102,427 (quantitative studies), 84 (qualitative studies)	Perinatal health outcomes (mental health); access, utilisation and experience of perinatal healthcare	The prevalence of clinically significant symptoms of postnatal depression and diagnosed postnatal depression for South Asian women who migrate to high-income countries is between 5 and 20%. This rate is likely to be under-reported because of a lack of specific sub-group analyses and studies on South Asian countries. Barriers to accessing healthcare need to be addressed including proficiency in English language, unfamiliarity with local services and lack of attention to mental health by healthcare providers
Pedersen et al. 2014 [24]	13	1969–2008	42,290,654 women including 6,102,663 migrant	Perinatal health outcomes (maternal mortality)	Migrant women in Western European countries have a doubled risk of dying during or after pregnancy when compared with indigenous-born women. A higher risk of death from direct causes suggests sub-standard obstetric care may be responsible for the majority of the excess deaths amongst migrant women
Schmied et al. 2017 [8]	15	1999–2015	256	Perinatal health outcomes (mental health); access, utilisation and experience of perinatal healthcare	Women who are migrants report higher levels of depressive symptoms, which can severely compromise mother-baby interaction and subsequent attachment relationships
Small et al. 2014 [33]	22	1990–2012	Sample sizes ranged from 6 to 432, with a total of 2498 migrant women	Access, utilisation and experience of perinatal healthcare	What migrant and non-migrant women want from maternity care is similar: safe, high-quality, attentive and individualised care, with adequate information and support. Migrant women were less positive about their care than non-migrant women. Communication problems and lack of familiarity with care systems negatively affected migrant women’s experiences, as did perceptions of discrimination and care which was not kind or respectful
Tobin et al. 2017 [20]	13	2004–2013	139	Perinatal health outcomes (mental health); access, utilisation and experience of perinatal healthcare	Migrant women with postnatal depression may lack understanding of their condition, are often isolated, alone, fear stigmatisation and risk being considered an unfit mother. Raising awareness with healthcare providers of the meaning of postnatal depression for migrant women is key to the provision of effective care
Villalonga-Olives et al. 2016 [29]	68	1994–2013	80,572,311 (6 studies no data reported)	Perinatal health outcomes (low birth weight)	The prevalence of low birth weight amongst migrants varies by the host country characteristics as well as the composition of migrants to different regions. The primary driver of migrant health is the migrant ’regime’ in different countries at specific periods of time. The ’healthy migrant effect’ in the USA is largely missing from Europe
Wikberg and Bondas 2010 [34]	40	1988–2008	More than 1160 women from more than 50 cultures	Experience of perinatal healthcare	*Alice in Wonderland* emerged as an overarching metaphor to describe intercultural caring in maternity care. There are specific cultural and maternity care features in intercultural caring: an inner core of caring consisting of respect, presence and listening, as well as external factors such as economy and organisation that affect intercultural caring. Legal status, power relationships and racism influence intercultural caring
Winn et al. 2017 [11]	19	1995–2015	Not reported	Access, utilisation and experience of perinatal healthcare	Three main meta-themes were developed: (1) Expectations Of Pregnancy As Derived From Home, (2) Reality Of Pregnancy In The Host Health Care System. These two themes were connected by our third meta-theme: Support
Wittkowski et al. 2017 [21]	16	1996–2011	337	Perinatal health outcomes (mental health); access, utilisation and experience of perinatal healthcare	Migrant mothers living in Western countries are subject to multifaceted and multifactorial stressors following the birth of their child, possibly making them more susceptible to developing postnatal depression and influencing their subsequent healthcare behaviour. These stressors are related to migration or being a migrant in a Western society as well as cultural influences which are harder to comply with as a migrant living in a different country, removed from their socio-cultural context. Social support appears to play an integral and mediating role for migrant mothers living in Western countries

^1.^ If the total sample size was not explicitly reported by the authors of the systematic review, then it was calculated from the table of included studies where possible

#### Perinatal mental health

Mental health, which included postnatal depression, antenatal depression, anxiety and post-traumatic stress disorder, was the most frequently reported outcome and was included in eleven systematic reviews; six were quantitative [5, 6, 16–19], three were qualitative [8, 20, 21] and two mixed methods [22, 23]. The reviews reported prevalence and risk factors for mental health disorders.

##### Prevalence of perinatal mental health disorders

All systematic reviews reporting prevalence data concluded that perinatal mental health disorders were more frequent in migrant women than in women from the host countries [5, 6, 16–19, 22]. Postnatal depression was the most frequently reported perinatal mental health outcome in the systematic reviews. Prevalence of postnatal depression amongst migrant women was reported as 11.2–60% [16], < 1–59% [5], 24–42% [18], 2.9–52% [22] and 20% (95% confidence interval (CI) 17–23%) [17]. Prevalence of antenatal depression amongst migrant women was reported to be 12–45% [5], and prevalence of any depressive disorder was 31% (95% CI 23.2–40%) [6]. There were also significantly increased associations with mental health disorders amongst migrant women compared with women from the host countries. Anderson et al. [5] reported that anxiety was increased in migrant women with non-English-speaking backgrounds, and post-traumatic stress disorder was 15% compared with 0% amongst non-migrant women. Nilaweera et al. [22] reported that odds ratios (ORs) for postnatal depression in their included studies ranged from 1.8–2.5 for migrant populations. Meta-analyses performed by Anderson et al. [5] and Falah Hassani et al. [17] also showed a higher odds of suffering from postnatal depression for migrant women compared to those from the host country (OR 1.56 (95% CI 1.31–1.86) and an adjusted OR (aOR) of 2.17 (95% CI 1.54–3.06 respectively)). When Falah Hassani et al. [17] adjusted for publication bias, the association decreased but remained significant (OR 1.67, 95% CI 1.12–2.30). Anderson et al. [5] also reported that associations differed for both antenatal and postnatal depression when stratifying the meta-analyses by country of study: antenatal depression USA (OR 0.71, 95% CI 0.51–0.99) and Canada (OR 1.86, 95% CI 1.32–2.62); postnatal depression USA (OR 0.87, 95% CI 0.59–1.28), Australia (OR 1.115, 95% CI 0.96–1.38) and Canada (OR 1.98, 95% CI 1.57–2.49).

##### Risk factors for the development of perinatal mental health disorders

Seven systematic reviews reported quantitative and qualitative evidence of factors associated with increased risk, or having a protective effect on perinatal mental health disorders [5, 6, 16–19, 22]. There were similarities between the systematic reviews, and results are reported under the themes of stress and support, adjustments to host country, pregnancy care and infant feeding, health status and history and socio-demographics.

- *Stress and support*. This was the most frequently and consistently reported risk factor for the development of mental health disorders amongst migrant women. Examples provided included emotional stress, a history of violence or abuse, having witnessed or experienced stressful life events and their premigration experience such as having migrated for political reasons or problems with the police or army in their home country [5, 6, 16–18]. Lack of social support and lack of family support were also reported to be important risk factors. There was a consistent pattern of low social support increasing the risk and good social support being protective against perinatal mental health disorders [5, 6, 17–19, 22]. Having no relatives or friends, a lack of emotional support from their spouse, being unmarried, having no partner, having migrated for marriage, marital adjustment problems and a lack of domestic decision-making power in relation to the child were all risk factors for perinatal mental health disorders amongst migrant women, whereas having a close relationship with their partner was reported to be protective [5, 6, 16, 17, 22].
- *Adjustment to host country*. The most commonly reported risk factors for perinatal mental health disorders were difficulties with the host country language [5, 6, 17, 19, 22] and being unfamiliar with local life [19]. Anderson et al. [5] reported inconsistent evidence in their included studies relating to the length of time resident in the host country, whereas other reviews reported that shorter duration of residence was a risk factor for perinatal mental health disorders [6, 17]. Fellmeth et al. [6] reported that adherence to traditional postpartum practices was protective against postnatal depression in migrant populations.
- *Pregnancy care and infant feeding*. Experience of perinatal healthcare including operative caesarean and instrumental delivery and poor satisfaction with support [6, 18] and also infant feeding experience including formula feeding and feeding problems [6, 17, 22] were risk factors for the development of perinatal mental health disorders reported by four systematic reviews [7, 17, 18, 22].
- *Health status and history*. The risk of perinatal mental health disorders was increased when migrant women perceived their overall health to be low [17, 19] or had a history of mental health disorders [6, 22]. Fellmeth et al. [6] reported ORs for postnatal depression to be between 24.9 and 29.7 when there was a personal or family history of depression.
- *Socio-demographics*. Risk factors included low income or socio-economic status, unemployment [5, 16, 17], low education [17], having a visible minority status [19] and primiparity [6]. Fellmeth et al. [6] also reported that maternal age > 30 years and < 25 years were risk factors for increased postnatal depression.

#### Mortality

Two systematic reviews reported data on maternal mortality (death of a woman during pregnancy, childbirth or in the first 42 days after delivery) [24, 25]. Pedersen et al. [24] reported the relative risk (RR) to be twofold amongst migrant women in Western European countries compared with women from the host countries (RR 2.00, 95% CI 1.72–2.33) and the absolute risk difference to be 9 additional maternal deaths per 100,000 deliveries per year for migrant women (95% CI 5.9–15.2). The strongest association was observed for direct causes of death amongst this population including hypertensive disorders (primarily preeclampsia and eclampsia), deep vein thrombosis and pulmonary embolism (RR 2.65, 95% CI 1.88–3.74) rather than indirect causes (unspecified) (RR 1.83, 95% CI 1.37–2.45) [24]. Gagnon et al. [25] included maternal mortality in a composite outcome for maternal health, although this article did not report the results for this outcome exclusively.

Three systematic reviews, all published in 2009, included offspring mortality [25–27]. Gissler et al. reported increased risks of stillbirth (RR 1.40, 95% CI 1.22–1.58), perinatal mortality (RR 1.35, 95% CI 1.26–1.45), neonatal mortality (RR 1.34, 95% CI 1.30–1.38) and infant mortality (RR 1.33, 95% CI 1.30–1.36) amongst migrant women in European countries compared with women from the host countries [27]. When the meta-analyses were restricted to migrants from non-European countries, the risk increased for stillbirths (RR 1.88, 95% CI 1.58–2.23) and slightly increased for perinatal, neonatal and infant mortality (RR 1.54, 95% CI 1.39–1.69; RR 1.40, 95% CI 1.36–1.44; RR 1.37, 95% CI 1.34–1.40 respectively). Conversely, migrant women in the USA had better outcomes than USA-born ethnic minorities (RR 0.77, 95% CI 0.63–0.65), demonstrating a healthy migrant effect. Adjustments for risk factors in the meta-analyses only accounted for a small proportion of the excess mortality risk [27]. Gagnon et al. [25] reported meta-analyses for feto-infant mortality (neonatal, infant mortality and spontaneous abortion). They found that Asian and North African migrant women had a significantly increased association with feto-infant mortality than women in the host country (aOR 1.29, 95% CI 1.02–1.63; aOR 1.25, 95% CI 1.10–1.41 respectively). There was no significant difference between majority-receiving-country women and European-born migrants (aOR 1.14, 95% CI 0.75–1.72) or Latin American-born migrants (aOR 1.02, 95% CI 0.76–1.39) [25]. The meta-analysis for African women showed the largest effect size, but this was not significant (OR 2.43, 95% CI 0.99–5.96) [25]. Note that these meta-analyses only included two or three studies for each country of origin and had high levels of heterogeneity. Bollini et al. [26] found an increased association between offspring mortality (including stillbirth, perinatal, neonatal, postnatal and infant mortality) and migrant women compared to women from the European host countries (OR 1.50, 95% CI 1.47–1.53). The authors hypothesised that pregnancy outcomes amongst migrant women were influenced by the degree of implementation of integration policies in the host countries, where a strong integration policy would be demonstrated by countries which had entrenched equality and social cohesion in their societies [26]. They carried out further meta-analyses adjusting for maternal age, parity and national level of implementation of integration policies and found the associations to be attenuated when there were strong implementation policies (aOR 1.25, 95% CI 1.17–1.34) compared with weak implementation policies (aOR 1.45, 95% CI 1.13–1.86); although the implementation of strong integration policies attenuated the association with offspring mortality, the difference in effect did not reach significance (*p* = 0.241) [26].

#### Mode of delivery

Three quantitative systematic reviews investigated mode of delivery amongst migrant women compared to women from host countries [7, 25, 28]. Gagnon et al. [25] reported that 40% of the 25 studies included in their review found operative modes of delivery (caesarean and operative vaginal) to be higher amongst migrant women; the remaining studies reported reduced operative mode of delivery outcomes for migrant women (20%), mixed results (12%) or no difference between migrant women and women from the host country (28%). Merry et al. also reported mixed results for caesarean delivery in their 2013 review [28]; associations between migrant women and caesarean varied by country of origin and by receiving country. The authors reported a significantly increased odds of caesarean amongst women migrating from former colonised Caribbean states (OR 1.91, 95% CI 1.37–2.66), South Asia (OR 1.28, 95% CI 1.22–1.35), the Philippines (OR 1.19, 95% CI 1.1–1.29) and Somalia (OR 1.13, 95% CI 1.02–1.26). Women migrating from Africa had increased odds of caesarean which differed according to receiving country: France (OR 2.22, 95% CI 1.92–2.58), Australia (OR 1.17, 95% CI 1.11–1.24), Canada (OR 1.34, 95% CI 1.08–1.67) and North/West Europe (OR 1.43, 95% CI 1.16, 1.77). However, these increased odds were not observed amongst women migrating from North Africa to Canada (OR 0.81, 95% CI 0.74–0.90) or France (OR 1.09, 95% CI 0.95–1.26). Similarly, women migrating from Latin America had significantly increased odds for caesarean in Norway (OR 2.41, 95% CI 1.79–3.23) and Canada (OR 1.43, 95% CI 1.29–1.59), but not in Southern Europe (OR 1.03, 95% CI 0.94–1.12). Odds for caesarean were significantly reduced or no different from those of women from receiving countries when women migrated from Vietnam (OR 0.68, 95% CI 0.66–0.71), Kosovo (OR 0.49, 95% CI 0.36–0.67), Russia/Baltic States (OR 0.75, 95% CI 0.66–0.85) and East Asia (receiving countries: Southern Europe (OR 0.59, 95% CI 0.47–0.73), USA (OR 0.73, 95% CI 0.71–0.75), and Australia, UK, Canada or Finland (OR 0.99, 95% CI 0.95–1.03)) [28]. The 2016 review of Merry et al. [7] was an update of the 2013 review and identified that migrant women from sub-Saharan Africa had higher caesarean section rates, whereas migrant women from Eastern Europe had lower rates than women in the host countries. Higher emergency caesarean deliveries were also reported for women migrating from Latin America, North Africa and the Middle East compared with women in the host countries [7].

#### Birth weight

Low birth weight (LBW) or small for gestational age (SGA) outcomes were reported by four reviews [19, 25, 26, 29] with contradictory results. A meta-analysis of LBW (< 2500 g) amongst migrant women residing in European countries showed significantly increased association compared with women in the European host countries (OR 1.42, 95% CI 1.42–1.44) [26]. There was a significant attenuation of LBW when analyses adjusted for age, parity and level of implementation of integration policies (*p* < 0.001); weak implementation resulted in an increased association (aOR 1.77, 95% CI 1.63–1.92) and strong implementation reduced the association (aOR 1.08, 95% CI 1.03–1.13), although the association remained significantly increased compared with results for non-migrant women [26]. Conversely, a meta-analysis of international data not restricted to women residing in Europe showed a reduced aOR for LBW and SGA amongst migrant women with borderline significance (aOR 0.92, 95% CI 0.85–1.00) [25]. Meta-analysis by migrant origin showed increased odds amongst women born in African and Asian countries and reduced odds amongst European, Latin American and North African-born women, although no sub-group meta-analysis reached statistical significance [25].

The paradoxical healthy migrant effect in relation to reduced risk of LBW and SGA was discussed by De Maio [19] and Villalonga-Olives et al. [29], although there were some reported inconsistent findings. The systematic review by De Maio [19] discusses how the patterns of reduced risk amongst migrants compared with women in the host countries are influenced by maternal socio-economic status, country of origin and maternal education, where migrant women with low levels of education have better outcomes and there is an increased risk of SGA and LBW amongst migrant women with higher education [19]. Villalonga-Olives et al. [29] discuss how the apparent healthy migrant effect in the USA (where migrant populations often have improved outcomes compared with non-migrant populations) is contrasted by the health inequalities in Europe, where the associations are reversed. The US studies show a reduced risk of LBW and SGA amongst Latina migrants, although this does not extend to Black and Puerto Rican migrants—who have increased risks—and Asian women show no difference in risk compared to women from the host country [29]. In contrast, there is a lack of a healthy migrant effect in Europe with the exception of studies from two countries, Spain and Belgium. However, data from these countries are also conflicting, showing that outcomes differ by migrant origins (e.g. increased risk amongst migrants from Morocco and Turkey) and also by the severity of outcome (e.g. women in host countries have a higher risk of moderate LBW, whereas migrant women have an increased risk of very LBW) [29].

#### Preterm birth

Three reviews reported preterm birth outcomes [19, 25, 26]. A meta-analysis by Bollini et al. [26] identified a higher odds of preterm birth (< 37 weeks gestation) amongst migrant women in Europe (OR 1.24, 95% CI 1.22–1.26). There was a significant attenuation when analyses adjusted for age, parity and level of implementation of integration policies (*p* < 0.001); weak implementation resulted in increased odds of preterm birth (aOR 2.88, 95% CI 2.50–3.32) and strong implementation policy decreased the odds (aOR 1.18, 95% CI 1.14–1.22) [26]. A meta-analysis reported by Gagnon et al. [25] found differences in risk of preterm birth by migrant origin. Compared with women in the host countries, there was a higher odds for migrant women from Asia (aOR 1.14, 95% CI 1.06–1.21) and Africa (aOR 1.29, 95% CI 1.04–1.60); a lower odds for Latina migrant women (aOR 0.83, 95% CI 0.72–0.95); and no difference for migrant women from Europe and North Africa [25]. De Maio [19] discusses the healthy migrant effect for preterm birth outcomes being influenced by maternal education and length of residence in the receiving country. Migrants with < 5 years residence had a lower prevalence of preterm birth compared with women in the host countries (4.7% vs 6.2%), and those residing > 15 years had the highest prevalence (7.4%) [19]. Further, a 5-year increase in length of residence significantly increased the odds of preterm birth amongst migrant women (aOR 1.14, 95% CI 1.10–1.19), which was potentially influenced by maternal stress and discrimination [19].

#### Congenital anomaly

Two reviews reported on congenital anomalies [25, 26]. Migrant women had a significantly increased risk of a pregnancy affected by a congenital anomaly compared to women in the host countries (OR 1.61, 95% CI 1.57–1.65). There was a significant attenuation of congenital anomalies when analyses adjusted for age, parity and level of implementation of integration policies (*p* < 0.001); when there was weak implementation, a significant increased association remained (aOR 1.20, 95% CI 0.95–1.52), whereas having a strong implement policy resulted in a significantly lower odds of congenital anomalies amongst migrant women (aOR 0.87, 95% CI 0.78–0.95) [26]. Gagnon et al. [25] combined congenital anomalies with other infant morbidity (such as neonatal intensive care unit (NICU) admission and low Apgar score) and found that 62.5% of the 16 studies included in their review reported worse outcomes for migrant women compared with women from the host country; no studies found this outcome to be better for migrant women.

#### Additional morbidities

Three reviews reported additional maternal or infant morbidities [19, 26]. Bollini et al. [26] investigated maternal postpartum haemorrhage, but no summary data were reported. De Maio [19] identified a healthy migrant effect for risk of placental dysfunction amongst women residing in Ontario, Canada, for < 5 years which was influenced by length of residence: the lowest odds were for women residing < 3 months (OR 0.53, 95% CI 0.47–0.61), which increased the longer the duration of residence (residing 48–59 months OR 0.82, 95% CI 0.77–0.87); however, the OR remained lower than that for women in the host country for all durations of residency. Gagnon et al. [25] had a composite outcome for maternal health (including but not limited to mortality, pregnancy-related morbidity, extended length of labour, episiotomy) and reported that 50% of their 32 included studies showed worse outcomes for migrant women, 21.9% showed better outcomes and the remaining studies were mixed or reported no difference. The authors also reported maternal and infant infections (including HIV, toxoplasmosis, sexually transmitted infections and rubella seronegativity) to be worse amongst migrant women in 63.6% of included studies and better in 9.1%; the remaining studies showed mixed results [25]. Bollini et al. [26] and Gagnon et al. [25] reported that admission to a NICU or special care was higher amongst offspring of migrant women (Gagnon et al. included NICU admission in their composite outcome for infant morbidities).

### Perinatal healthcare access and experiences amongst women who are migrants (including asylum seekers and refugees)

Twenty systematic reviews reported access to, and experience of, perinatal healthcare amongst migrant women; 11 were qualitative [8, 9, 11, 16, 20, 21, 30–34], 5 were quantitative [6, 18, 19, 25, 35] and 4 were mixed methods systematic reviews [10, 22, 23, 36]. Results are summarised in Table 2, and a narrative summary is presented of the themes and sub-themes relating to: (1) access to and utilisation of perinatal healthcare and (2) experience of perinatal healthcare.

1. Access to and utilisation of perinatal healthcare
  Sixteen systematic reviews reported data relating to access or utilisation of perinatal healthcare [8–11, 16, 19–23, 25, 30, 32, 33, 35, 36]. All systematic reviews reported that access to perinatal care, including routine care and specialist care such as mental health support for postnatal depression, was worse amongst migrant women. Heaman et al. [35] reported that 86% of the 29 studies included in their review showed inadequate prenatal care for migrant women compared with women in the host countries, with 15 studies reporting large effect sizes (aORs > 2.0). Gagnon et al. [25] reported that prenatal care was worse amongst migrant women compared with women in the host countries in 58.3% of their 12 included studies, and no studies reported care to be better amongst migrant women. Barriers to accessing care were consistent across all systematic reviews and are summarised here under the themes of structural and organisational barriers, social barriers and personal and cultural barriers.
  - Structural and organisational barriers
    Ten systematic reviews reported unfamiliarity with local healthcare provision, culture and systems as a barrier [8, 10, 19–22, 30, 32, 33, 36]. Issues included a lack of knowledge and awareness of services and support on offer, a lack of information provision about how to get support, difficulties with navigating healthcare systems, managing bureaucracy and a lack of information about regular appointments and check-ups which resulted in missed appointments. Ten systematic reviews reported language barriers to accessing perinatal healthcare [10, 11, 19–23, 32, 35, 36] including proficiency in being able to verbally communicate with health professionals, access to translators and understanding written communication. Physician availability, long waiting lists for services, especially those specialising in migrant care, a lack of postnatal follow-up and perceptions that health services did not want to take migrant women were additional structural and organisational barriers reported in three systematic reviews [20, 23, 36].
  - Social barriers
    Nine systematic reviews reported social barriers to accessing care which centred on the competing priorities of *real life worries* [21] that migrant women faced such as poverty, safe housing, employment and caring for their other children [8, 10, 11, 20, 21, 31, 32, 35, 36]. Financial constraints were frequently reported including a lack of health insurance, cost of care and wider poverty issues such as having no phone, childcare or transport [8, 10, 11, 20, 21, 32, 35, 36]. Further social barriers included having an unplanned pregnancy, being single and maternal education level [31, 35].
  - Personal and cultural barriers
    Six systematic reviews reported personal and cultural barriers to accessing services [8, 9, 20, 21, 23, 36]. Three reviews focussed on accessing perinatal mental health services [8, 20, 21] and reported a reluctance amongst migrant women to talk about mental health, a lack of cultural acceptability to seek help, beliefs about women’s strength and self-coping, the fear of labelling, stigma and alienation in some cultures, beliefs that depression was not a real health condition, that health professionals and services were for physical health, a lack of understanding of the condition and associated terminology and fears of having their child removed. Additional barriers reported were a lack of culturally appropriate therapists and services available [20, 36] and a preference for female health professionals due to religious reasons and the intimacy of body areas during pregnancy [9, 23]. However, the preference for female providers was negated in emergency situations, and the competency of the health professional was considered more important than gender [9].
2. Experience of perinatal healthcare
  Twelve systematic reviews reported data relating to migrant women’s experiences of perinatal healthcare [8, 10, 11, 16, 20–22, 30, 32–34, 36]. There were some consistent experiences reported in the systematic reviews, and these are summarised under the themes of negative communication and discrimination, relationships with health professionals, cultural clashes and the receipt of clinical perinatal healthcare.
  - Negative communication and discrimination
    Language barriers and having to rely on translators had an impact on communication experiences [8, 10, 11, 30, 32–34]. Systematic reviews also reported themes of insensitive and hurtful communication, perceptions of racism, cultural stereotyping and discriminatory interactions between migrant women and health professionals [10, 33, 34, 36]. Small et al. [33] reported that the migrant women felt that care was not kind or respectful and that they were less likely to be spoken to with respect, understanding and in a way they could comprehend.
  - Relationship with health professionals
    The interpersonal relationship between migrant women and health professionals was reported to be an important influence on experience of perinatal care. A positive experience resulted from health professionals who were kind and friendly and who listened to the woman’s concerns [10, 32]. However, the majority of data related to struggles with relationships and a lack of connection; migrant women were less likely to describe health professionals positively than women in the host countries [20, 30, 32, 33]. There was a common theme of migrant women feeling rushed during interactions with health professionals [8, 10, 32, 36], misunderstandings with health professionals and a lack of confidence to express concerns or ask questions [11, 30].
  - Cultural clashes
    Ten systematic reviews reported a lack of cultural knowledge and sensitivity in their experiences of perinatal healthcare [8, 10, 11, 20, 21, 30, 32–34, 36]. The majority of these clashes stemmed from differences between cultural, religious and traditional beliefs and practices and Western biomedical approaches to perinatal healthcare. The systematic reviews reported that migrant women lacked understanding about Western medicine and care, felt pressure to adapt and were labelled as non-compliant if they resisted Western approaches in favour of traditional practices [11, 20, 21, 30, 32, 34, 36].
  - Clinical perinatal healthcare
    Nine systematic reviews reported migrant women’s experiences of clinical perinatal healthcare including breastfeeding support, decision making about care and Western approaches to medicine and technology [10, 16, 20–22, 30, 32, 33, 36]. Higginbottom et al. [36] reported some positive experiences amongst migrant women relating to provision of breastfeeding support in hospital; however, the remaining data relate to negative experiences of care. The reviews reported that migrant women were less positive about the care they received and reported that health professionals discussed their care with them less frequently than with women in the host countries, especially relating to mental healthcare needs [16, 22, 33, 36]. The reviews also reported that migrant women did not feel involved in decision making about their care or did not feel they had options [10, 33, 36]. There were reports of poor experiences of care and pain management amongst migrant women who had female genital mutilation (FGM) [30, 33, 36], too much focus on technological and procedural approaches to care and childbirth [30, 32] and an over-reliance on prescription of medications which were culturally or religiously inappropriate rather than access to supportive care (e.g. counselling for depression) [20, 21, 30, 32].

### Results specific to women with asylum seeker or refugee status

Twenty-two of the included systematic reviews either explicitly reported results relevant to women with asylum seeker or refugee status, or they cited studies where the participants were exclusively women with asylum seeker or refugee status [5–11, 14, 16, 18–20, 23, 27–34, 36]. In total, the reviews cited 54 studies, although there was some overlap in the original studies relevant to asylum seekers and refugees included in the systematic reviews and used to inform the analyses (Additional file 5); data from 43 unique studies were cited by these 22 systematic reviews. Eight systematic reviews use primary data that view asylum seekers and refugees as separate sub-groups [5, 6, 16, 18, 19, 23, 30, 36]. Five systematic reviews [7, 9–11, 34, 36] combine asylum seekers and refugees as a sub-group of the migrant population. Seven systematic reviews [8, 20, 27–29, 32, 33] use primary data solely involving refugees. Two systematic reviews [14, 31] use primary data looking at asylum seekers only. Seven systematic reviews [17, 21, 22, 24–26, 35] do not distinguish asylum seekers and refugees from other types of immigrants in their analysis.

The data reported for asylum seekers and refugees were limited, and the majority of detailed data came from qualitative studies on women’s access to and experiences of perinatal healthcare. The results table is summarised in Additional file 6, and a narrative summary is presented for perinatal health outcomes and healthcare access and experiences amongst women with asylum seeker or refugee status.

#### Perinatal health outcomes amongst women with asylum seeker and refugee status

Fourteen systematic reviews reported perinatal health outcomes for asylum seeker and refugee populations [5–7, 14, 16, 18–20, 23, 27–30, 36]. Perinatal health results are presented for perinatal mental health, offspring mortality, mode of delivery, birth weight, preterm birth and additional morbidities. Perinatal mental health was reported most frequently by the systematic reviews. No data specific to asylum seekers or refugees were reported for maternal mortality or congenital anomaly outcomes.

##### Perinatal mental health amongst women with asylum seeker or refugee status

Nine systematic reviews reported data for asylum seekers and refugees [5, 6, 16, 18–20, 23, 30, 36] citing 11 original studies [37–47].

##### Prevalence of perinatal mental health disorders

Five reviews cited data from Stewart et al. [37] which found that rates of postnatal depression were significantly higher amongst women with refugee and asylum seeker status (25.7% and 31.1% respectively) compared with women in the host country of Canada (8.1%, *p* = 0.008). They also found a significantly increased odds of scoring 10 or more on the Edinburgh Postnatal Depression Scale for refugees (OR 4.80, 95% CI, 1.57–14.69) and asylum seekers (OR 3.06, 95% CI, 1.06–8.82) [5, 16, 18, 19, 23]. Similar rates were reported in a systematic review by Fellmeth et al. [6]; 37.3% of refugees and 41.8% of asylum seekers living in Canada experienced symptoms of depression, somatisation or anxiety and significantly increased odds for the prevalence of any depressive order (OR prevalence 0.25, 95% CI 0.21–0.29) (data from Gagnon et al. [48]). Increased prevalence for post-traumatic stress disorder were reported, where asylum-seeking women had the highest prevalence (48.2% above the cut-off), followed by refugees (33.8%) and migrants (15%) [5, 6] (data from Gagnon et al. [48]). Higginbottom et al. [36] reported that of 50 refugee mothers who received a home visit at 4 months postpartum, 26 were found to have symptoms of postpartum depression (data from Merry et al. [39]). Data from original studies exclusively on women with asylum seeker or refugee status [40–44, 47] were used in a meta-synthesis by Balaam et al. [30] and contributed to the findings that stress and low self-esteem were common, and that women had mental health problems such as depression, feelings of loneliness and isolation and expressed sadness, vulnerability and anxiety together with severe nausea.

##### Risk factors for the development of perinatal mental health disorders

Three systematic reviews reported risk factors for the development of perinatal mental health disorders specifically relevant to asylum seekers and refugees [6, 18, 19]. Fellmeth et al. [6] reported data from Matthey et al. [45] which showed statistically significant associations between anxiety and the number of premigration traumatic events experienced or witnessed, but no association with anxiety or post-traumatic stress disorder and history of living in a refugee camp prior to resettlement. Collins et al. [18] and De Maio [19] presented data from Stewart et al. [37] which found that refugees and asylum seekers had significantly lower social support than women in the host country of Canada (*p* < 0.001), including support from family, friends, groups and systems, as well as personal, emotional and instrumental social support. Tobin et al. [20] also reported that women who were refugees attributed their depression to social factors such as family problems or economic hardship rather than biological factors (data from Edge [46]).

##### Offspring mortality amongst women with asylum seeker and refugee status

Two systematic reviews [14, 27] reported offspring mortality amongst women who were refugees using data from nine original studies [40, 49–56]. Gissler et al. [27] reported that in European studies women who were registered refugees or originated from refugee source countries at the time of arrival (including Africa, sub-Saharan Africa, Romania, Kosovo and Russia) had a significantly increased risk of stillbirth (RR 2.01, 95% CI 1.41–2.06), early neonatal mortality (RR 2.77, 95% CI 1.85–4.13) and perinatal mortality (RR 1.71, 95% CI 1.41–2.06) compared to women in the host countries of Norway, Sweden, Ireland and the Netherlands. However, women from Vietnamese backgrounds had lower mortality than women in the host country of Norway [27]. Evidence from the former Yugoslavia showed that women who were refugees had increased risk of early neonatal mortality (RR 3.66, 95% CI 1.92–6.99) and perinatal mortality (RR 3.07, 95% CI 2.05–4.62) but no difference in risk of stillbirth (RR 1.19, 95% CI 0.56–2.50). Deaths attributed to congenital anomalies, pregnancy complications or intrauterine growth restriction were similarly distributed amongst refugees and women in the host country.

##### Live birth and abortion

There were additional data relevant to offspring mortality for women with asylum seeker and refugee status that were not reported in the data for migrant women. Hadgkiss and Renzaho [14] reported that asylum seekers had a higher incidence of sexual assault, unwanted pregnancies and induced abortion-to-live birth ratio compared with women in the host countries (1:2.5 vs 1:7.5) (data from Goosen et al. [55], Kurth et al. [40] and Rogstad and Dale [56]). Asylum seekers with longer duration of stay (compared with those arriving in the previous 6 months) had a lower live birth and abortion rate [14] (data from Goosen et al. [55]).

##### Mode of Delivery amongst women with asylum seeker and refugee status

Three systematic reviews [7, 14, 28] reported caesarean delivery for refugee and asylum seeker women using data from four original studies [38, 40, 57, 58] with conflicting results. Merry et al. [28] and Hadgkiss and Renzaho [14] reported data from two studies [40, 57] which found no significant difference in caesarean delivery rates amongst asylum seekers compared to native-born women (OR 0.93, 95% CI 0.74–1.17) (data from Gagnon et al. [57]). However, Merry et al. [7] reported that refugees and asylum seekers were at a reduced risk of an emergency caesarean compared with economic and student migrants (data from Gagnon et al. [38]) but an increased risk compared with women in the host country of Canada (data from Kandasamy et al. [58]).

##### Birth weight amongst women with asylum seeker and refugee status

Two systematic reviews [14, 29] reported data for low birth weight (LBW) and intrauterine growth retardation using data from five original studies [40, 49, 59–61]. Villalonga-Olives et al. [29] reported no difference in LBW between refugee populations in Ireland or undocumented Latina migrants in the USA and women in the host countries (data from Kelaher and Jessop [61] and Lalchandani et al. [49]). However, Somali refugees in Belgium, Canada, Finland, Norway and Sweden had lower rates of LBW compared with women in the host countries (data from Small et al. [59]). Hadgkiss and Renzaho [14] reported prevalence of intrauterine growth restriction to be one of the most prevalent outcomes amongst women who were seeking asylum, 7% of the population (data from Kurth et al. [40]; no comparison data were reported for women in the host countries).

##### Preterm birth amongst women with asylum seeker and refugee status

Two systematic reviews [14, 27] reported preterm birth amongst women who were refugees using data from two original studies [40, 54]. Hadgkiss and Renzaho [14] reported premature labour to be one of the most prevalent outcomes in women seeking asylum at 15% of the population (data from Kurth et al. [40]), and Gissler et al. [27] reported that women who were displaced from the former Yugoslavia had higher preterm rates than women in the host country (data from Nedic et al. [54]).

##### Additional morbidities amongst women with asylum seeker and refugee status

Two systematic reviews [14, 30] reported additional maternal morbidities and data from six original studies [40–42, 55, 56, 62]. The additional morbidity outcomes reported for women with asylum seeker and refugee status (eclampsia, obstetric haemorrhage and maternal infections) are similar to those reported for migrant women (placental dysfunction, postpartum haemorrhage and maternal infection). There were additional data reported explicitly for women with asylum seeker and refugee status that were not reported for migrant women (including asylum seekers and refugees) showing increased risk of severe acute maternal morbidity (SAMM), gestational diabetes, anaemia and uterine rupture. There was a lack of data explicitly for women with asylum seeker and refugee status and offspring infection and admission to special care units. Hadgkiss and Renzaho [14] reported that asylum seekers faced a range of complex obstetric issues including bleeding, gestational diabetes, anaemia, 4.5 times higher incidence of SAMM than the general obstetric population (31.0 vs 6.8 per 1000 births), uterine rupture (15 vs 8.4%) and eclampsia (27.5 vs 9.1%); but lower incidence of obstetric haemorrhage (42.5 vs 63.3%) (data from Kurth et al. [40], Goosen et al. [55], Rogstad and Dale [56], Van Hanegem et al. [62]). Baalam et al. [30] reported poor health amongst women with asylum seeker and refugee status which caused complications for the women and the newborn babies, including infected wounds, HIV and hepatitis (data from Kennedy and Murphy-Lawless [41] and McLeish [42]).

#### Healthcare access and experiences amongst women with asylum seeker and refugee status

Twelve systematic reviews reported access to or experience of perinatal healthcare amongst women with asylum seeker and refugee status [8–11, 20, 23, 30–34, 36]. The sub-themes presented are the same as the results for migrant women. However, there are additional results within the sub-themes for asylum seekers and refugees that were not present, or not as detailed, for migrant women.

##### Access to perinatal healthcare amongst women with asylum seeker and refugee status

The barriers to accessing care are summarised here under the themes of structural and organisational barriers, social barriers and personal and cultural barriers.

1. Structural and organisational barriers
  Seven systematic reviews reported structural or organisational barriers for women with asylum seeker and refugee status to access perinatal healthcare [10, 11, 23, 30, 31, 33, 36] including data from 15 original studies [39–44, 47, 57, 63–69]. Results relating to limited ability to speak the language of the host country or understand the verbal or written information provided [10, 23, 30, 33] were similar to the results for the overall migrant population, as were challenges navigating, and a lack of familiarity with, the healthcare systems and inadequate information about what support services exist [10, 11, 30, 31, 33, 36]. Additional data relevant to asylum seekers and refugees included a lack of knowledge about availability of support services which led to feelings of social isolation [36]. There were assumptions amongst asylum seekers and refugees that they would have to pay for perinatal healthcare when they were entitled to free care [10, 31] and mistrust of healthcare professionals who were perceived to be a threat to the emotional and physical safety of asylum seekers who did not engage with antenatal care [31]. Higginbottom et al. [23] also reported that learning the host country language was not a priority for women, and that the men in the household attended language classes while the women stayed at home.
2. Social barriers
  Six systematic reviews reported social barriers to accessing perinatal healthcare [8, 10, 11, 30, 32, 36] including data from 12 original studies [39–44, 64, 66, 70–73]. Many of the social barriers to accessing or continuing with perinatal healthcare were similar to those for migrant populations such as a lack of finances, transport, issues with housing and a lack of family and friend networks [8, 10, 11, 30, 32, 36]. However, these difficulties were described in the systematic reviews to be particularly challenging for women with asylum seeker or refugee status due to temporary and uncertain status, not being permitted to work in their host countries and the impact of these factors on available resources and having a ’normal life’ [8, 36]. For example, Higginbottom et al. [36] describes postnatal refugees skipping meals because of a lack of resources, and Balaam et al. [30] reported that some types of accommodation for refugees and asylum seekers are restricted by fixed mealtimes which imposed practical challenges with flexibility to attend appointments. Mengesha et al. [10] reported that home visits by refugee health nurses were positively received, and Balaam et al. [30] reported that childbirth was a critical milestone towards a better social status, and that the baby represented a new beginning and a health resource.
3. Personal and cultural barriers
  Five systematic reviews reported personal and cultural barriers to accessing perinatal healthcare [9, 20, 23, 30, 32] including data from 11 original studies [40–44, 67, 71, 74–77]. The systematic reviews reported similar results to those for migrants in relation to a lack of cultural understanding of postnatal depression and a preference for female health professionals. Further context was provided on gender preference for asylum seekers and refugees. Aubrey et al. [9] reported that higher rates of caesarean deliveries amongst Syrian refugee women resulted from avoidance in seeking antenatal care due to the lack of female health professionals and the fact that only 5 out of 18 African refugee women in the USA would accept care from a male health professional. However, these findings were in conflict with other studies in their review which reported that African refugee women accessing obstetric care in Australia, and Somali women in the USA, would accept care from a male health professional in an emergency [9].

##### Experience of perinatal healthcare amongst asylum seekers and refugees

The themes identified in the systematic reviews around experience of care related to negative communication and discrmination, relationship with health professionals, cultural clashes, and clinical perinatal care are summarised below.

1. Negative communication and discrimination
  Seven systematic reviews reported negative communication and discrimination data for asylum seeker and refugee women [10, 11, 23, 30, 33, 34, 36] reporting data from 12 original studies [39–44, 63, 64, 66–68, 73]. There were similar negative communication experiences to the results for migrant women, including reliance on interpreters and experience of discrimination. However, these negative experiences were more widely represented in the data specific to women with asylum seeker and refugee status than for general migrant populations. Balaam et al. [30] reported that refugee and asylum-seeking women were less willing to state their needs and wishes. Data relating to reliance on interpreters represented an inadequacy of service provision leading to delayed care, women’s reliance on body language and facial expressions to communicate, their needs not being met and the women being unable to express their concerns. There was a reported need for more consistent professional interpreting support for women with asylum seeker or refugee status including integrated services, continuity of competent interpreters and improving of health professionals’ knowledge of when interpreting services are required [10, 23, 30, 33, 36].
  The systematic reviews reported that the most vulnerable women with asylum-seeking or refugee status had the most difficult situation and negative encounters with health professionals including openly racist and discriminatory care, cultural stigma, disrespect, hostility, stereotyping and being treated as ’primitive people’ [10, 30, 33, 34]. These experiences are demonstrated in a quote from an included study reported in the systematic review by Wikberg and Bondas [34]: “An African woman asked for help when she got an infection but was not met with respect: She looked at me like this and said, ’You are OK’.. . She said to another midwife, ’These Africans. .. they come here, they eat nice food, sleep in a nice bed, so now she doesn’t want to move from here!’ . .. When she said this I didn’t say anything, I just cried… she doesn’t know me, who I am in my country. And the other midwife said ’What’s wrong with them, these Africans?’ and some of them they laughed” (data from McLeish [42]). Women reported that these interactions were influenced by skin colour, their language ability and communication problems, and that they wanted supportive, non-discriminatory care [30, 33].
2. Relationship with health professionals
  Four systematic reviews reported data on the relationships between health professionals and women with asylum seeker and refugee status [10, 11, 30, 33] from 10 original studies [40–44, 47, 63, 64, 68, 70]. There were similar findings to the results for overall migrant populations in relation to the importance of a supportive relationship with health professionals, negative experiences such as feeling health professionals were too busy and a lack of confidence to discuss their issues with health professionals [10, 30, 33]. Positive interactions were experienced when health professionals had respect for practices from the country of origin or were of the same ethnicity or religion, and positive support increased confidence in asking questions and acceptance of the new healthcare system and practices [11, 30].
3. Cultural clashes
  Five systematic reviews reported cultural clashes in perinatal healthcare experience amongst women with asylum seeker and refugee status [10, 11, 30, 32, 33], reporting data from five original studies [63, 64, 66, 70, 71]. All data specific to women with asylum seeker and refugee status duplicate the findings of the overall migrant women, such as tensions between feeling the need to adapt to host country medical practices and women’s preferences for traditional cultural or religious practices. No new findings were identified in the data specific to women with asylum seeker and refugee status.
4. Clinical perinatal healthcare
  Six systematic reviews reported issues with the clinical perinatal healthcare amongst women with asylum seeker and refugee status [10, 20, 30, 32, 33, 36] reporting data from 15 original studies [39–44, 47, 57, 63, 64, 66, 68, 71, 73, 78]. There were some similarities with the results for migrant women relating to negative experiences amongst women with asylum seeker and refugee status, health professionals showing a lack of knowledge and sensitivity relating to FGM, women receiving poor explanations of care and lack of discussion of options, a lack of assessment and referrals for postnatal depression, an over-reliance on technology and Western practices which lacked cultural sensitivity [10, 30, 32, 33, 36]. Additional findings in the data for women with asylum seeker and refugee status include the following: outcomes being better amongst women who were able to exhibit resilience and adjust and change their cultural beliefs; disappointment and lack of preparation for the lack of practical postnatal help and support; recommendations for advocacy or link-worker schemes; and the need for culturally appropriate health education materials on labour and delivery and health professional training on Somali refugee women’s culture, traditions, values and expectations [30, 33]. The systematic review by Tobin et al. [20] reported discrepancies on the topic of support groups for postnatal depression in their included studies; one study reported limited use for refugee women who preferred individual therapy due to privacy, confidentiality and a cultural stigma related to the condition, whereas another study found that social networking and support groups were important in facilitating help seeking and the healing process.

## Discussion

This systematic review of systematic reviews aimed to summarise the existing evidence base of perinatal health outcomes and perinatal healthcare amongst women with the status of asylum seeker and refugee. Although all included reviews incorporated data for women with asylum seeker or refugee status in order to be eligible for inclusion, the data reported specific to this population were limited. Only one included systematic review was exclusively focussed on asylum seekers, and the remaining data for asylum seeker and refugee women were grouped with those for heterogeneous migrant populations or other vulnerable women in the evidence syntheses. We found that a number of perinatal health outcomes were worse for migrant women than women in the host country, including mental health disorders, maternal mortality, preterm birth and congenital anomalies. The qualitative and quantitative evidence specifically relevant to women with asylum seeker and refugee status suggests that they have worse outcomes and experiences compared to the evidence from wider migrant populations (including asylum seekers and refugees) and to women in the host country, particularly relating to complex obstetric issues (e.g. SAMM, uterine rupture, eclampsia), mental health, offspring mortality, sexual assault and unwanted pregnancy, FGM, infectious disease and anaemia. However, similarities in population risk between asylum seekers, refugees and wider migrant populations were observed for some perinatal health outcomes, such as caesarean deliveries. The healthy migrant effect was reported in some of the systematic reviews, particularly relating to LBW where the risk was similar to or better than that for women in the host countries. This was reported by some authors as being an explanation for better outcomes. The evidence suggests that the healthy migrant effect is context-specific and does not translate across all migrants from all countries of origin or receiving countries. Systematic reviews reported a healthy migrant effect amongst specific populations (primarily Latina migrants in the USA) where outcomes tended to be improved compared with women in the host country, either native-born or other migrant groups. However, health inequalities were reported amongst migrant populations from other origin and/or host countries and amongst refugee and asylum seekers who, for certain outcomes, fared worse than either other migrant women or women from the host country. The heterogeneity between migrant, asylum seeker and refugee population leads us to further question the appropriateness of grouping migrant populations in research, practice and policy. Combining populations may mask the true differences in perinatal health outcomes and care requirements, and without these data the development of targeted interventions to prevent adverse outcomes is hindered.

Despite the lack of systematic reviews exclusively focussing on women with asylum seeker and refugee status, there were some data on these populations available to explore perinatal health issues amongst these groups of women. The majority of the literature which specifically focussed on women with refugee and asylum seeker status explored access to and experience of perinatal healthcare. These data showed similar barriers to access and use of perinatal healthcare as for wider migrant populations. However, additional depth of data relevant to asylum seeker and refugee women included social isolation resulting from barriers to care, mistrust of health professionals and financial concerns and poverty; the latter barriers were particularly challenging due to the inability to work and temporary and uncertain status of residency. Women’s experiences of care also showed similarities to those for wider migrant populations but with apparently increased challenges with language and communication barriers and more widespread experience of racism, discrimination, stigma and stereotyping in encounters with perinatal healthcare services and professionals.

This systematic review has several strengths, particularly the comprehensive search strategy. We searched 12 databases, using a search strategy developed with an information scientist with expertise in database searching. The search strategy was developed and pre-tested using MEDLINE, then refined and retested until we were confident that it was both sensitive and specific. We also searched the reference lists of all of the included systematic reviews and implemented citation searching. These supplementary searches identified a further eight systematic reviews, which demonstrates the importance of supplementing rigorous database searches with additional search strategies. This is particularly important when searching for qualitative or observational evidence, which can be limited when using databases alone, and is a recommended approach to search strategies in the Meta-analysis Of Observational Studies in Epidemiology (MOOSE) guidelines [15]. The quality of the included reviews was judged to be either moderate or high; no review was considered to be of poor quality. However, supplementing electronic database search strategies was only carried out by authors of 20 included systematic reviews, which suggests that there may be some element of publication bias in the existing evidence syntheses. Only 21% of studies explored publication bias, which may compound bias from combining heterogeneous migrant population definitions in the analysis. A further strength includes screening and data extraction carried out independently by two researchers. We used a validated quality assessment tool to assess the quality of each included review. However, despite our use of a comprehensive search strategy, we cannot be certain that we have retrieved all relevant reviews, as our searches were restricted to English language reviews.

The main limitation of this systematic review of systematic reviews relates to data availability in the existing reviews. We had set out to consider selected pregnancy care and perinatal health outcomes specifically for women who were asylum seekers or refugees, but this couldn’t be undertaken in depth as the existing evidence identified often did not allow for these sub-groups to be analysed separately. Despite the large number of studies of migration and perinatal health, there was limited evidence available for a number of pregnancy outcomes including pregnancy complications such as obstetric haemorrhage, maternal infections, maternal mortality and congenital anomalies, and although all reviews included data for women with asylum seeker and refugee status, there were limited results specific to this population. This highlights that although studies on migrant health have increased in recent years, certain maternal and offspring health outcomes remain under-researched, which limits the conclusions that can be drawn. There were also limited data exploring the risk factors for developing adverse outcomes between different migrant populations. The majority of risk factor data related to the development of mental health disorders, although these data were not stratified by the specific migrant population in question, which challenges the interpretation and application into routine care.

A review of systematic reviews will naturally result in overlapping data from multiple reviews incorporating the same original study data. We have addressed this in our review relating to the analysis of data specific to asylum seekers and refugees, detailing the number of unique studies that contributed to the results and listing these studies in Additional file 6: Asylum and Refugee Data, and by reporting the data explicitly for women with asylum seeker and refugee status separately from the results for migrant women including asylum seekers and refugees. Due to the primary focus of this review being on asylum seekers and refugees, and the volume of data relating to migrant populations (which included asylum seekers and refugees), it was not feasible to go into this level of detail for overlapping studies for this population; this is a limitation. However, we do not believe that the identification of overlapping studies included in the systematic reviews that were not explicitly related to asylum seeker and refugee populations would have added to the interpretation of results, given that the major challenge to interpretation was in the grouping of these heterogeneous populations.

Our systematic review of systematic reviews suggests a number of areas that warrant further research. There is limited evidence for women with asylum seeker and refugee status on particular perinatal outcomes such as maternal mortality, obstetric complications such as haemorrhage and infections and congenital anomalies. There is also a paucity of research into the potential causal pathways between migrant statuses and adverse health outcomes. Migrants, asylum seekers and refugees are specific populations; investigating health outcomes for these groups when they are combined presents challenges for furthering research as well as for policy and practice. When it was possible to compare migrant populations including asylum seekers and refugees with asylum seekers and refugees in this review, we were able to demonstrate some similar findings for particular health outcomes but also different and worse outcomes which are masked when groups are combined. We were unable to analyse data specific to asylum seekers and specific to refugees. We only identified one systematic review specific to asylum seekers, which suggests that further research is needed. Our systematic review specifically searched for systematic reviews on asylum seekers and refugees, but studies on other groups of vulnerable women, e.g. undocumented and migrant workers, are also needed. The development of effective interventions to support these women will not be possible if heterogeneous groups continue to be combined for research.

Our findings on the healthcare experiences of women with asylum seeker and refugee status have implications for practice. Interactions with healthcare professionals were far from optimum, with communication, discrimination and stereotyping reported. Current UK [79] and Australian [80]) guidelines share the common recommendations of health professionals needing to understand the specific needs of these groups of women; that a variety of means should be used to support women; and that there is a need to inform women of antenatal services and how to use them. Given the findings of this review on risks of maternal mental health and obstetric complications, the provision of mental health services and facilitation of timely access to antenatal care is essential for this population of women. Healthcare commissioners should also have a clear understanding of local needs so that appropriate services can be planned [79]. Implementing these recommendations into practice and providing culturally specific training for health professionals have the potential to reduce some of these negative experiences for women and also for health professionals.

## Conclusions

This systematic review of systematic reviews demonstrates that women with asylum seeker and refugee status have worse perinatal health outcomes, including mental health, offspring mortality and preterm birth, compared to women from other migrant groups. Further research is warranted on particular perinatal health outcomes, e.g. maternal mortality, as well as on understanding potential causal pathways. Access, use and experience of perinatal healthcare were also reported to be far from optimal. This represents inequalities for migrant women, especially those with asylum seeker or refugee status. Improvements in care are urgently needed to increase access and enhance the experience amongst these vulnerable populations. There is an urgent need for the inclusion of unambiguous definitions of migrant groups to be used in research and for analysis to be stratified by migrant status and other migration indicators, e.g. country of origin and length of time spent in the host country. The results of this review support the need for future research on perinatal health which can make specific recommendations for policy and practice.

## Additional files

Additional file 1: PRISMA checklist. Completed PRISMA reporting checklist. (DOC 58 kb)

Additional file 2: Database searches. A summary of the database search terms used in the search strategy. (DOCX 17 kb)

Additional file 3: Sample data extraction and quality appraisal. A completed example of the data extraction and quality assessment templates used in the systematic review. (DOCX 89 kb)

Additional file 4: Description of included systematic review populations. Table providing a description of the populations included in the systematic reviews that were included in this systematic review of systematic reviews. (DOCX 25 kb)

Additional file 5: Quality assessment of included systematic reviews. Table of scoring for each included systematic review against quality assessment criteria. (DOCX 17 kb)

Additional file 6: Summary of results and data sources relevant to women with asylum seeker or refugee status directly referred to by the included systematic reviews. Table providing an overview of data relating to asylum seekers and refugee women in the included systematic reviews. (DOCX 35 kb)

## Abbreviations

aOR: Adjusted odds ratio
CI: Confidence interval
FGM: Female genital mutilation
JBI: Joanna Briggs Institute
LBW: Low birth weight
NICU: Neonatal intensive care unit
OR: Odds ratio
PICOS: Population Intervention Comparator Outcome Study design
RR: Relative risk
SAMM: Severe acute maternal morbidity
SGA: Small for gestational age
UK: United Kingdom
UNCHR: United Nations High Commissioner for Refugees
USA: United States of America
