# Supplementary material for: Perinatal health outcomes and care among asylum seekers and refugees: a systematic review of systematic reviews
Source: BMC Med. 2018 Jun 12;16:89. doi: 10.1186/s12916-018-1064-0 (PMC5996508; doi:10.1186/s12916-018-1064-0)
Supplement: Supplementary file 2 — Database searches. A summary of the database search terms used in the search strategy. (DOCX 17 kb) [file 12916_2018_1064_MOESM2_ESM.docx]

**Additional file 2. Database searches**

**OVID MEDLINE searches 1946 to July week 2017 and Embase 1974 to 2017 July.**

1.pregnanc* outcom*.mp.

2.refugee*.mp.

3.1 and 2

4.refugee* and antenata* access).mp.

5. (refugee* and postnat* care).mp.

6. (maternal health and refugee*).mp.

7. asylum seeker preg* outcome.mp.

8. (refugee preg* and outcome).mp.

9. (refugee and maternity services).mp.

10. (refugee and perinata* care).mp.

11. (asylum seeker and pregnancy outcome).mp.

12. (asylum seeker and post partum care).mp.

13. 2 and 8

14. (asylum seek* and maternity).mp.

15. (refugee health and utilisation).mp.

16. 13 and 14

**Scopus searches 19/07/17**

1. (pregnancy asylum seeker or refugee women).

2. (refugee* and preg*).

3. (refugee* and antenal* care).

4. refugee*.

5. (refugee and postnatal*).

6. (refugee and post partum*).

7. 1 and 2

8. 1 and 4

9. 1 and 2 or 4

10. 1 and 3 or 4

11.Pregnancy or preg/

12.Perinata/ or antena*

**Proquest searches 19/07/17**

1.(refugee* and preg*).mp. [mp=ti,

2. (refugee* and antenal* care).mp.

3. refugee*.mp. and antenatal/

4. (refugee and postnatal*).mp.

5. (refugee and post partum*).mp.

6. (refugee and perinata*).mp.

7. systematic review*.mp.

8. 1 and 7

9. refugee delive/

10. refugee labour ward.mp. and birth/

11. refugee birth/

12. refugee access preg*.mp.

13. refugee matern* care.mp.

14. (refugee* and preg*).mp.

15. (refugee* and antenal* care).mp.

16. refugee*.mp. and antenatal/

17. (refugee and postnatal*).mp

18. (refugee and post partum*).mp.

19. (refugee and perinata*).mp.

20. 7 and 19

21. 8 or 20

**Google scholar 17/07/17**

1. Systematic review pregnancy AND asylum seeker

2. systematic review preg*AND refugee

3. systematic review access OR utilisation maternity services AND asylum seeker OR refugee

4. systematic review asylum seeker AND access maternity service, Asylum seekers AND pregnancy,

5.systematic review asylum seekers OR refug/ AND pregnan/

6.systematic review AND maternal* health AND asylum* seekers

7. systematic review perinat/ care AND asylum seeker OR refug/

8.systematic review prenatal care AND asylum seeker or refugee

9 systematic review pregnancy outcome AND asylum seekers OR refugees

**Science Direct 18/07/17**

1.Systematic review AND asylum seekers

2. pregnancy OR refugee

3. systematic review asylum seekers OR refugee AND pregnancy outcome

4. systematic review on asylum seekers pregnancy and inequality

**Web of science 21/07/17**

1.Systematic review AND asylum seekers OR refugees*

2.asylum seeker AND pregnan*

3. refugee AND pregnancy

4. Refugee/ and access*

5. matern* service and refug*

6. refugee and utilise

7. refugee AND utilisat*

**PubMed 20/07/17**

1. Systematic review AND asylum seeker

2. refugee AND pregnancy

3. systematic review asylum seekers OR refugees AND pregnancy

4. Systematic review AND asylum seekers and maternal health

5. asylum seekers AND pregnancy

6. systematic review AND asylum seekers AND pregnancy refugee OR asylum seeker AND pregnant

**Cochrane database of systematic review 16/07/17**

1. Asylum seekers AND pregnancy OR cost of care

2. Asylum seeker AND delivery

3. Asylum seeker OR refugee women

4. perinatal health AND asylum seekers

5. systematic review pregnancy AND refugee OR asylum seeker

6. maternal health AND asylum seeker OR refugee

**Prospero 20/07/17**

1.systematic review refugee or asylum seekers

2. refugee or asylum seekers

**JBI 20/07/17**

1.Asylum seeker OR refugee

2. refugee AND pregnancy

3. maternal health AND refugee

4. Asylum seeker, refugees

**CINAHL 16/07/17**

1.Asylum seeker AND pregnancy

2.Refugee AND pregnancy

3.Refugee OR asylum seeker AND pregnant

4.Refugee AND maternal health

5.Refugee AND access matern care

6.Refugee AND utilise antenat*

7.Antenatal AND asylum

8.Perinat* AND refugee

9.Postpar* and refugee*

10.Postpart/ AND asylum*

11.Maternity AND refugee
