# Supplementary material for: Perinatal health outcomes and care among asylum seekers and refugees: a systematic review of systematic reviews
Source: BMC Med. 2018 Jun 12;16:89. doi: 10.1186/s12916-018-1064-0 (PMC5996508; doi:10.1186/s12916-018-1064-0)
Supplement: Supplementary file 3 — Sample data extraction and quality appraisal. A completed example of the data extraction and quality assessment templates used in the systematic review. (DOCX 89 kb) [file 12916_2018_1064_MOESM3_ESM.docx]

**Additional file 3: Sample data extraction and quality appraisal**

| **Data Extraction carried out by** (name): Heather Brown and Hayley Coleman | |
| --- | --- |
| **Date that data extraction was carried out**: 4 August 2017 | |
| **Review study details** (Include full reference for review paper, authors, title, journal title etc):  Alhasanat, D. and J. Fry-McComish (2015). "Postpartum depression among immigrant and Arabic women: literature review." Journal of Immigrant and Minority Health 17(6): 1882-1894. | |
| **Aim and objectives/focus of the review:** | |
| **Review type** (Please tick relevant box). | Quantitative with meta-analysis  x  Quantitative with narrative  Qualitative  Mixed methods  Other: Please provide details: |
| **Review Aims and Objectives:**  as defined by the author. If the aims and objectives are unclear/not stated then state ‘unclear’ | The purpose of this literature review is to identify the prevalence and risk factors for PPD among immigrant women in industrialized countries and compare it with prevalence and risk factors for PPD among Arab women in their home countries. |
| **Participants**: | Asylum seekers only  Asylum seekers and refugees  Refugees only  X  Migrant including asylum seekers and/or refugees |
| **Sample** definition (for study participants included in the review): | Please state sample definition as given by authors:  In the US, an immigrant is an alien or a person with no US citizenship at birth. This includes lawful permanent residents, refugees, asylum seekers, persons on certain temporary visas, and the unauthorized |
| **Inclusion/exclusion criteria** (i.e. the inclusion/exclusion criteria for the systematic review, not the inclusion/exclusion criteria of the individual studies included in the review): | Inclusion:  Inclusion criteria were:  (a) original research, (b) risk factors for PPD, (c) sample consists of adult women over 18 years of age, and (d) immigrant women or adult Arab women are essential in the sample.  Exclusion:  a) antenatal depression and major depression-focused research studies,  (b) papers whose samples were selected on the basis of ethnicity rather than immigration status, and (c) books |
| **Outcomes included in search strategy**  (list the outcomes/topics that were **searched for** in the review, e.g. mental health, women’s experiences of care) | keywords of postpartum depression/postnatal depression AND risk factors/predictors AND immigrant/Arab/Middle Eastern. |
| **Methods: Search Details** | |
| **Date range** of search (for the review) | 1990-2013 |
| **Search strategy** (include databases searched and supplementary searches carried out) | Four databases were used: MEDLINE, CINAHL, PsycINFO, and PubMed.  review of references |
| **Restrictions**:  (Provide details of restrictions to the search strategy if stated. E.g. language) | The search in each database was limited to English language, peer-reviewed publications, academic journals, and published between 1990 and 2013. |
| **Appraisal rating** of included studies | |
| Author’s appraisal rating reported?  **Appraisal tool used:** | Yes No  X |
|  | n/a |
| **Summary of quality of included studies** as described by the authors of the review: | n/a |
| **Key results:** | |
| Total number of studies included | 26 (14 for immigrant women and 12 for Arabic women in their countries.) |
| Publication date range of included studies | Immigrant studies 1998-2013 |
| Total sample size of pregnant women reported | 9089 |
| Countries of included studies | Studies for immigrant women:  6 Studies from Canada  3 Studies from Australia  2 studies from Taiwan  3 studies from the US |
| Description of population of women **included** in the studies (relating to asylum seeker or refugee status) | The overall definition of immigrant women was consistent across the 14 studies. Twelve studies identified immigrant women as women who were born outside the country (Canada, Taiwan, Australia, and US) or born overseas in a non-english speaking country. Two studies  made some classification within the immigration status, such as immigrants, asylum seekers, and refugee women |
| **Summary quantitative results** (key findings for the **overall population** included):  Meta-analysis:  Narrative (if no meta-analysis presented): | **PPD Prevalence Among Immigrant Women**  All 14 studies found that immigrant women had higher rates of probable postpartum depression than native women. PPD was measured using screening tools rather than diagnostic tools. Among the Canadian studies that reported prevalence, the prevalence of PPD symptoms among immigrant women ranged from 11.2 to 37 %. [8,  31, 34, 36].  In the Australian studies that reported prevalence, immigrant women were more likely than native women to score C13 on the EPDS, indicating probable depression [32, 35]. In the Taiwan studies, higher  prevalence of PPD was reported among immigrant women, which ranged between 25.5 and 41.1 % [29, 30]. In the US studies, the prevalence of PPD symptoms among immigrant Hispanic women was 54–60 % [23–25].  **Risk Factors for PPD Among Immigrant Women**  Lack of social support [28–34, 37], immigration stress [23, 32, 36, 37] stressful life events [23, 30, 31], and lack of emotional support from the spouse [28, 31, 35, 36] emerged as common risk factors for PPD among immigrant women across studies. Low family income and socioeconomic status were also found as risk factors for developing  PPD among immigrant women [29, 31, 34]. Access to health care services in the postpartum period was examined in two studies, and found that immigrant women were less likely to be able to obtain care for emotional health problems or to be asked about their feelings by health care providers [8, 35]. |
| **Detailed quantitative results** (detailed results **explicitly** relating to **asylum seekers and refugee** populations):  Meta-analysis:  Narrative (if no meta-analysis presented):  (If no results are presented explicitly relating to just asylum seeker and refugee populations then state “no data presented”) | (**Ref 33** Stewart D, Gagnon A, Saucier J, Wahoush O, Dougherty G.  Postpartum depression symptoms in newcomers. Can J Psychiatry.  2008;53(2):121–4.)  Stewart et al. Immigrants (35.1 %), asylum seekers (31.1 %), and refugees (25.7 %) were more likely than Canadian-born (8.1 %) women to score C10 on EPDS  Only one study examined violence, and that study found violence against the woman during pregnancy or in the postpartum period was a risk factor for developing PPD symptoms among immigrant women in Canada [37]. |
| **Summary qualitative results** (key findings for the **overall population** included):  Themes and subthemes: | n/a |
| **Detailed qualitative results** (detailed results **explicitly** relating to **asylum seekers and refugee** populations):  (If no results are presented explicitly relating to just asylum seeker and refugee populations then state “no data presented”) | n/a |
| **Overall conclusions** (As stated by authors - for the **overall population** included) | In this review, there were some similarities in the risk factors for PPD among immigrant women and Arabic women in their country of birth: lack of social support, stressful life events, lack of emotional support from the partner, history of antenatal depression, and marital dissatisfaction.  Immigration stress and lack of access to health care services were found among immigrant women. Lack of social support was more predominant in studies on immigrant women. |
| **Specific conclusions for asylum seeker and refugee populations** (As stated by authors) | Immigrant and refugee women experience many risk factors that may affect their mental and physical health such as marginalization and minority status, pre-migration experiences, painful memories, socioeconomic constraints, poor physical health, and difficulty adapting to new countries and cultures [56, 57]. |
| **Reference list screened** | Yes No  X |
| **References to follow up as potential systematic reviews** to screen against inclusion criteria (list citation numbers or authors) | None |

**JBI Critical Appraisal Checklist for Systematic Reviews and Research Syntheses**

|  | **Yes** | **No** | **Unclear** | **Not applicable** |
| --- | --- | --- | --- | --- |
| 1. Is the review question clearly and explicitly stated? |  |  |  |  |
| 1. Were the inclusion criteria appropriate for the review question? |  |  |  |  |
| 1. Was the search strategy appropriate? |  |  |  |  |
| 1. Were the sources and resources used to search for studies adequate? |  |  |  |  |
| 1. Were the criteria for appraising studies appropriate? |  |  |  |  |
| 1. Was critical appraisal conducted by two or more reviewers independently? |  |  |  |  |
| 1. Were there methods to minimize errors in data extraction? |  |  |  |  |
| 1. Were the methods used to combine studies appropriate? |  |  |  |  |
| 1. Was the likelihood of publication bias assessed? |  |  |  |  |
| 1. Were recommendations for policy and/or practice supported by the reported data? |  |  |  |  |
| 1. Were the specific directives for new research appropriate? |  |  |  |  |
