# Supplementary material for: Perinatal health outcomes and care among asylum seekers and refugees: a systematic review of systematic reviews
Source: BMC Med. 2018 Jun 12;16:89. doi: 10.1186/s12916-018-1064-0 (PMC5996508; doi:10.1186/s12916-018-1064-0)
Supplement: Supplementary file 4 — Description of included systematic review populations. Table providing a description of the populations included in the systematic reviews that were included in this systematic review of systematic reviews. (DOCX 25 kb) [file 12916_2018_1064_MOESM4_ESM.docx]

| **Systematic Review Author, year** | **Systematic review definition of included migrant, asylum seeker or refugee population** | **Country of origin of migrants, asylum seekers or refugees** | **Included host countries** | **Comparison group** |
| --- | --- | --- | --- | --- |
| Alhasanat and Fry-McComish 2015 | In the US, an immigrant is an alien or a person with no US citizenship at birth. This includes lawful permanent residents, refugees, asylum seekers, persons on certain temporary visas, and the unauthorized | China and Vietnam, Mexico, Indonesia, Hispanic, Philippines, Turkey, Arabic  countries, Anglo-Celtic, and Western women | USA | Arab women in their home countries |
| Anderson *et al.* 2017 | Migrants in this study, refers to everyone living outside of their country of birth, including refugees, asylum-seekers, economic migrants. | Not specified however one of the included studies specified looking at Chinese migrant | Canada, USA, Australia, Taiwan, China | Non-migrant (born in study country) |
| Aubrey *et al.* 2017 | This study focussed on women that have migrated from the Middle East, Africa, South Asia to Europe and North America | Middle East, Africa, and South Asia | Europe and North America | Women migrating from different religious-cultural environment |
| Balaam *et al.* 2013 | Migration is motivated by several factors, such as economic improvement, family reunion, and the seeking of refugee status, and takes a range of forms including ‘illegal’, undocumented, forced, free and controlled. However, all these forms of migration are important factors in the  erosion of traditional boundaries between languages, cultures, ethnic groups, and nation states. | Somali, Eritrean,  Chinese, Scotland, Sudan, Turkey, Middle East, Asia, East European, Afghanistan, Congo, Rwanda, Yugoslavia | Sweden, UK, Switzerland, Norway, Ireland, Greece | None |
| Bollini *et al.* 2009 | Described immigrants as persons living in a country other than the one in which they are a citizen of. | Africans, Arabs, Asian and Pacific Islanders,  Bangladeshi, Caribbean  Chinese, East Africans  Eastern Europe, Indians, Indochinese  Irish, Italians Latinos  Moroccans  Mediterranean  Middle East, North  Africa. Turkish  Western European,  North American,  West African, West Indians and Yugoslavs | European countries specifically UK, Norway, Spain, Denmark, Netherlands, Belgium, France, Italy, Austria, Germany, and Switzerland | Native women in Europe |
| Collins *et al.* 2011 | Immigrant women in this study excludes second generation immigrant and women who were selected solely on the basis of ethnicity rather than immigration. | South Asian, Vietnam, Turkey, Philippines, Arabic countries, Anglo-Celtic, and Western. | Canada, Australia, and Taiwan | Native-born  Women. |
| De Maio, 2010 | Immigrants to Canada | Not specified, however data from South Asian and African women were analysed in the Ontario diabetes database | Canada | Native born population |
| Downe *et al.* 2009 | This study included papers exploring antenatal care experiences, attitudes and/ or beliefs of high risk, marginalised minority pregnant women receiving inadequate care living in resource-rich countries. | Pakistani/Bangladeshi, Somali, Arab American, African American, Mexican | UK, USA, and Canada | Not specified |
| Falah-Hassani *et al.* 2015 | Immigrants were described as a person who came to live permanently in a foreign country. | China or Vietnam, Taiwanese, South Western Sydney, Pakistani, Sweden Indonesian, Turkish and Filipino | Canada USA, UK, Sweden, Norway, Switzerland, Australia, Taiwan | Non-immigrant  women |
| Fellmeth *et al*. 2017 | Migrants constitute a heterogeneous group and reasons for migrating are multifaceted, involving a complex interplay between factors within and beyond individuals’ control. Seeking better opportunities for education, employment and health, and securing better means to support family are important factors, along with more acute drivers such as natural disasters, violence and conflict.  Focussed on women from low and middle income countries who have resettled in any region, including other low and middle income countries.  Migrants include refugee and asylum seekers as well as economic migrants | Latin America, Southeast Asia, Philippines, Cambodian and Pakistan | North America, Europe, Australasia | Non-migrant women |
| Gagnon *et al*. 2009 | This study includes all women who had migrated (studies were excluded if international cross-border movement was unlikely). | Mexico, Middle-East North Africa, sub-Sahara, South American, Asian Finns in Sweden, Surinamese, West  Indian, Turkish, Moroccan, Ghanaian, other non-Dutch.  North Africans, South Americans, South/East  Asian and Pacific Islanders Pakistanis | USA, UK, France, Italy, Norway, Australia, Sweden, Spain, Other Europe/Canada | Receiving-country women. |
| Gissler *et al.* 2009 | Authors used the definitions provided by the original studies which they grouped as:  (1) country of birth or foreign-born: any indicator which required data on country of birth to define it;  (2) ethnicity: an undefined term used by authors, which could include ethnicity, ethnic group, ethnic mix, race;  (3) nationality: a commonly undefined term which could include national origin, citizen, citizenship, ‘extra-community’ (i.e. extra-European Union);  (4) ‘foreigner’: an undefined migrant in which it was unclear what had been used to define the term;  (5) language: any term requiring data on language to define it (for example ‘non-English speaking’);  (6) refugee: the term used to include those who left home unwillingly and/or had been in resettlement camps; and  (7) Immigrant status: including terms such as ‘undocumented’, ‘illegal’, or ‘irregular’. | Africa, Romania, Kosovo, Russia, Sub-Saharan Africa, Somalia Vietnamese, Philippines Turkey, Morocco Ethiopia, Eritrea Pakistan, Sri Lanka, India, Bangladesh, Morocco, Algeria, Tunisia | Spain , Italy, Belgium, Sweden, Croatia, Ireland, The Netherlands, Serbia, Norway, UK, USA, and Australia | Women born in receiving countries |
| Hadgkiss and Renzaho, 2014 | According to the 1967 Protocol of the 1951 Convention Relating to the Status of refugees, a refugee is any person owed protection outside the country of their nationality or birth because they have a well-founded fear that they will be persecuted because of their race, religion, nationality, political opinion or membership of a particular social group. When that person’s claim for protection is not approved and/or still being assessed, they are referred to as asylum seekers. | Not specified | Switzerland, England, Australia, Netherlands, New Zealand, Ireland, Wales, USA, Scotland, Canada | Not specified |
| Heaman *et al*. 2013 | Defined a migrant woman as a woman who left her usual place of residence to establish a permanent new residence in a Western industrialized country. This was collapsed into a single group | French Caribbean, Indian Ocean, Southern Europe,  Northern Africa, Sub-Saharan Africa, Asian South/East Asia, Puerto Rico and Mexico | France, Netherlands, USA, UK, Finland, Germany, and Belgium | Receiving-country women |
| Higginbottom *et al.* 2015 | Defined an immigrant as a person who has settled permanently in Canada. This includes economic migrants, skilled workers, temporary foreign workers, documented and undocumented residents, refugee claimants, refugees, asylum seekers and students | South Asian, Vietnamese Canadian, Euro Canadian,  Nigeria, Mexico, India  Colombia and St. Vincent, Pakistan, Bangladesh and Sri Lanka, India and Columbia, Spanish, Portuguese, Indo-Canadian, Somali, West Asian/Arab | Canada | Non-immigrants |
| Higginbottom *et al.* 2014 | This study includes paper describing immigrant women’s experience of maternity care. | Vietnamese, South Asian, Cambodian, Laotian, Punjabi | Canada | Canadian residency |
| Higginbottom *et al.* 2012 | This study used the definition of an immigrant provided by the Canadian Council for Refugees (2010): ‘a person who has settled permanently in another country.’ However, the term is hugely heterogeneous and includes economic migrants and skilled workers, temporary foreign workers, documented and undocumented residents, refugee claimants, refugees, asylum seekers and students. | Punjabi, Chinese, South Asian, Hong Kong, Somali and Vietnamese women | Canada | Not specified |
| Mengesha *et al.* 2016 | In general, the Australian Government has three immigration pathways: migration for skilled migrants; family migrants; and  a humanitarian program for those in humanitarian need (e.g. refugees, asylum seekers, women at risk).  In the context of this paper, the term ‘culturally and linguistically diverse’ (CALD) includes those born outside Australia and whose first language is not English | Afghanistan, Africa, China, Palestine, Lebanon, Syria, Iran and Jordan, Vietnam, China, Japan, Korea and Philippines, Ethiopia, Egypt, Lebanon, Iraq, Jordan, Saudi Arabia, Syria, Lebanon, Jordan and Turkey | Australia | Compared with  Australian born women, |
| Merry *et al.* 2013 | Migrants were described as immigrants, refugees, asylum-seekers, undocumented migrants and others with temporary or irregular statuses | Migrants in Western industrialized countries (North Africa /West Asia (Middle East) East Asia, Latin America Caribbean (non-Hispanic) Eastern Europe, Southern Europe | Europe, USA, Canada, Australia, Israel | Receiving country-  born women |
| Merry *et al.* 2016 | International migrants were described as individuals who have moved from one country to establish themselves temporarily or permanently in another country | Sub-Saharan Africa, South Asia, Eastern European, Vietnam Latin America and North Africa/Middle-East | Europe, USA, Canada, Australia, Israel, Japan | Non- migrant  Women. |
| Nilaweera *et* *al.* 2014 | This study was focussed around South Asia migrant women who gave birth in a country other than their homeland are poorly understood | Spanish, Chinese, South Asians, Indians, Australia, Sri Lankans, French, Afghanistan, Bangladesh, Bhutan, India, Maldives, Nepal, Pakistan and Sri Lanka | Australia, Canada, USA, UK, Norway | Not specified |
| Pedersen *et al*. 2014 | Migrant women were defined by a different country of origin or nationality than the receiving country women | Scotland, Ireland,  Europe, USSR &  Mediterranean,  African, Caribbean,  Southern Asia, Pakistani, Indian, Bangladeshi, Black African Caribbean other Asians | US  England, Netherlands, Germany, France, Spain, and Switzerland | Non-migrant women |
| Schmied *et al.* 2017 | The term migrant is defined by the International Organization for Migration (IOM) as ªany person who is moving or has moved across an international border or within a State away from his/her habitual place of residence, regardless of 1) the person's legal status; 2) whether the movement is voluntary or involuntary; 3) what the causes for the movement are; or 4) what the length of the stay.  This broad definition therefore includes social and economic migrants, humanitarian migrants, as well as those seeking asylum. | Central America, the Caribbean; South America; Asia, Africa and the Middle East. | Australia Canada, USA, UK | Not specified |
| Small *et al* .2014 | For the purposes of this review, we define immigrant women as those women not themselves born in the country in which they are giving birth. | Vietnam, China, Cambodia, Laotia, Thailand, Korea, Phillipinees, Turkey, Somali, South Asia, Eritrea and Sudan, South America including Puerto Rico | Australia Sweden, UK, USA, Canada | Non-immigrant women |
| Tobin *et al.* 2017 | Refugee and/or immigrant women  No explicit definition published, authors discussed displaced persons:  “Globally, we are witnessing the highest level of displaced people in history, with an unprecedented 65.3 million displaced persons on record, 21.3 million of whom are refugees (United Nations High Commissioner for Refugees, 2016).” | Not specified | USA, Canada, UK | Not specified |
| Villalonga-Olives *et al*. 2016 | Not clear | Asia, Latina, Samoan, US Hispanic/Latino, Korean, Japanese, Mexican, Caribbean Chinese, Indian, European, African, non-Spanish speaking Caribbean, Yugoslavia, Somalia, Lebanon, Pakistan, Turkey, Algeria, Morocco, Central Africa, Northern Africa and Middle East, Eastern Europe, Asia, Latin America and Tunisia | US/Europe  Italy, England, Wales, Belgium, France, Norway, Sweden, Denmark, Spain, Finland, Ireland, Greece  Australia, USA | Compared to the native-born |
| Wikberg and Bondas, 2010 | Culture in this study refers to ‘‘a pattern of learned but dynamic values and beliefs that gives meaning to experience and influences the thoughts and actions of individuals of an ethnic group’’ | Mexican-American, Eritrea, Somalia, Sudan, China, Hong Kong, Taiwan, Ethiopian, Japanese, Hmong, Turkish, Cambodian, Lao,  Vietnamese, Caribbean, African, Southeast Asian,  Mediterranean, Algeria, Congo, Angola, Nigeria, Puerto Rico, Dom. Rep.,  Somalia, Iraq, Pakistani, Thai, Bangladeshi, Indian,  Portugal, Kuwait, Malaysia, Singapore | Finland, USA, Canada, Australia, Israel, Japan, South Africa, Scandinavian countries | Not specified |
| Winn *et al*. 2017 | Immigrant or refugee women | Asia, Africa, and South/Central America (Hispanic) | North America  (USA and Canada) | Non-immigrant  women |
| Wittkowski *et al.* 2017 | The inclusion criteria refer to immigrants which includes recent immigrants and the more settled (e.g., second and third generation) descendants of immigrants, who, in the studies, might be referred to as ethnic minorities. | Hmong, Jordan, Middle East, Bangladeshi, Indian, other Asian countries, Caribbean, Japan, Pakistani, Egypt,  Haiti, Latina | UK USA, Canada, Australia | Non-immigrant mothers |
