# Supplementary material for: Perinatal health outcomes and care among asylum seekers and refugees: a systematic review of systematic reviews
Source: BMC Med. 2018 Jun 12;16:89. doi: 10.1186/s12916-018-1064-0 (PMC5996508; doi:10.1186/s12916-018-1064-0)
Supplement: Supplementary file 5 — Quality assessment of included systematic reviews. Table of scoring for each included systematic review against quality assessment criteria. (DOCX 17 kb) [file 12916_2018_1064_MOESM5_ESM.docx]

**Additional file 5: Quality assessment of included systematic reviews**

| **Author, year** | **Quality assessment question** | | | | | | | | | | | **Total score** | **Quality** |
| --- | --- | --- | --- | --- | --- | --- | --- | --- | --- | --- | --- | --- | --- |
|  | **1** | **2** | **3** | **4** | **5** | **6** | **7** | **8** | **9** | **10** | **11** |  |  |
| Alhasanat and Fry-McComish, 2015 | Yes | Yes | Yes | No | No | No | Unclear | Yes | No | Yes | Yes | **6** | **moderate** |
| Anderson *et al*. 2017 | Yes | Yes | Yes | Yes | Yes | Yes | Unclear | Yes | Yes | Yes | Yes | **10** | **high** |
| Aubrey *et al.* 2017 | Yes | Yes | Yes | Yes | No | Unclear | Unclear | Yes | No | Yes | Yes | **6** | **moderate** |
| Balaam *et al*. 2013 | Yes | Yes | Yes | Yes | Yes | Yes | Unclear | Yes | No | Yes | Yes | **9** | **high** |
| Bollini *et al.* 2009 | Yes | Yes | Yes | No | Yes | Unclear | Unclear | Yes | No | Yes | Yes | **7** | **moderate** |
| Collins *et al*. 2011 | Yes | Yes | Yes | No | No | No | Unclear | Yes | No | Yes | Yes | **6** | **moderate** |
| De Maio, 2010 | Yes | Yes | Yes | Yes | No | No | Unclear | Yes | No | Yes | Yes | **7** | **moderate** |
| Downe *et al.* 2009 | Yes | Yes | Yes | Yes | Yes | Yes | Yes | Yes | No | Yes | Yes | **10** | **high** |
| Falah-Hassani *et al*. 2015 | Yes | Yes | Yes | Yes | Yes | Yes | Yes | Yes | Yes | Yes | Yes | **11** | **high** |
| Fellmeth *et al*. 2017 | Yes | Yes | Yes | Yes | Yes | Yes | Yes | Yes | No | Yes | Yes | **10** | **high** |
| Gagnon *et al*. 2009 | Yes | Yes | Yes | Yes | Yes | Yes | Unclear | Yes | No | Yes | Yes | **9** | **high** |
| Gissler *et al.* 2009 | Yes | Yes | Yes | Yes | No | No | Yes | Yes | No | Yes | Yes | **8** | **high** |
| Hadgkiss and Renzaho, 2014 | Yes | Yes | Yes | Yes | Yes | Yes | Yes | Yes | No | Yes | Yes | **10** | **high** |
| Heaman *et al*. 2013 | Yes | Yes | Yes | Yes | Yes | Yes | Yes | Yes | No | Yes | Yes | **10** | **high** |
| Higginbottom *et al.* 2015 | Yes | Yes | Yes | Yes | Yes | Yes | Yes | Yes | No | Yes | Yes | **10** | **high** |
| Higginbottom *et al*. 2014 | Yes | Yes | Yes | Yes | Yes | Yes | Yes | Yes | No | Yes | Yes | **10** | **high** |
| Higginbottom, *et al.* 2012 | Yes | Yes | Yes | Yes | Yes | Yes | Yes | Yes | No | Yes | Yes | **10** | **high** |
| Mengesha *et al.* 2016 | Yes | Yes | Yes | Yes | No | No | Yes | Yes | No | Yes | Yes | **8** | **high** |
| Merry *et al.* 2013 | Yes | Yes | Yes | Yes | Yes | Yes | No | Yes | Yes | Yes | Yes | **10** | **high** |
| Merry *et al.* 2016 | Yes | Unclear | Unclear | No | No | No | Unclear | Yes | Unclear | Yes | Yes | **4** | **moderate** |
| Nilaweera *et al*. 2014 | Yes | Unclear | Yes | NO | Yes | Yes | Unclear | Yes | No | Yes | Yes | **7** | **moderate** |
| Pedersen *et al.* 2014 | Yes | Yes | Yes | Yes | No | No | Yes | Yes | Yes | Yes | Yes | **9** | **high** |
| Schmied *et al*. 2017 | Yes | Yes | Yes | Yes | Yes | Yes | Yes | Yes | No | Yes | Yes | **10** | **high** |
| Small *et al.* 2014 | Yes | Yes | Yes | NO | No | No | Yes | Yes | No | Yes | Yes | **7** | **moderate** |
| Tobin *et al*. 2017 | Yes | Yes | Unclear | Yes | Yes | Yes | No | Yes | No | Yes | Yes | **8** | **high** |
| Villalonga-Olives *et al.* 2016 | Yes | Yes | Yes | NO | No | No | No | Yes | No | Yes | Yes | **6** | **moderate** |
| Wikberg and Bondas, 2010 | Yes | Yes | Yes | NO | Unclear | Unclear | Unclear | Yes | No | Yes | Yes | **6** | **moderate** |
| Winn *et al.* 2017 | Yes | Yes | Yes | Yes | Yes | Yes | Yes | Yes | Yes | Yes | Yes | **11** | **high** |
| Wittkowski *et al*. 2017 | Yes | Yes | Yes | Yes | Yes | Yes | Unclear | Yes | Yes | Yes | Yes | **10** | **high** |
| **Total “yes” score for each question** | **29** | **27** | **27** | **21** | **18** | **17** | **14** | **29** | **6** | **29** | **29** |  | |
| **Percent scored “yes” for each question** | **100%** | **93%** | **93%** | **72%** | **62%** | **59%** | **48%** | **100%** | **21%** | **100%** | **100%** |  |  |

Notes: 1) Is the review question clearly and explicitly stated?; 2) Were the inclusion criteria appropriate for the review question?; 3) Was the search strategy appropriate?; 4) Were the sources and resources used to search for studies adequate?; 5) Were the criteria for appraising studies appropriate?; 6) Was critical appraisal conducted by two or more reviewers independently?; 7) Were there methods to minimize errors in data extraction?; 8) Were the methods used to combine studies appropriate?; 9) Was the likelihood of publication bias assessed?; 10) Were recommendations for policy and/or practice supported by the reported data?; 11) Were the specific directives for new research appropriate?. For total score 1 is given if yes otherwise it is zero. For quality: Low quality is 0-3. Moderate quality is 4-7. High quality is 8-11.
