# Supplementary material for: Perinatal health outcomes and care among asylum seekers and refugees: a systematic review of systematic reviews
Source: BMC Med. 2018 Jun 12;16:89. doi: 10.1186/s12916-018-1064-0 (PMC5996508; doi:10.1186/s12916-018-1064-0)
Supplement: Supplementary file 6 — Summary of results and data sources relevant to women with asylum seeker or refugee status directly referred to by the included systematic reviews. Table providing an overview of data relating to asylum seekers and refugee women in the included systematic reviews. (DOCX 35 kb) [file 12916_2018_1064_MOESM6_ESM.docx]

**Additional file 6. Summary of results and data sources relevant to women with asylum seeker and refugee status directly referred to by the included systematic reviews**

| **Systematic review author, year^1^** | **Topic Area** | **Key results and/or conclusions for asylum seeker and refugees reported in the systematic reviews** | **References for studies within the included reviews reporting data specifically for asylum and refugee populations^2^** |
| --- | --- | --- | --- |
| Alhasanat and Fry-McComish. 2015 | Perinatal health outcomes (mental health) | Postpartum depression was higher among asylum seeker and refugee women than Canadian-born. Refugee women experience many risk factors that may affect their mental and physical health such as marginalization and minority status, pre-migration experiences, painful memories, socioeconomic constraints, poor physical health, and difficulty adapting to new countries and cultures. | - Stewart *et al.* (2008). Postpartum depression symptoms in newcomers. *The Canadian Journal of Psychiatry*, *53*(2), 121-124. |
| Anderson *et al.* 2017 | Perinatal health outcomes (mental health) | Asylum-seeking and refugee women had an increased risk of depression and high prevalence of post-traumatic stress disorder symptoms. | - Stewart *et al.* (2008). Postpartum depression symptoms in newcomers. *The Canadian Journal of Psychiatry*, *53*(2), 121-124. - Gagnon AJ *et al.* (2013) International migration to Canada: the post-birth health of mother and infants by immigration class. *Soc Sci Med* 76: 197-207 |
| Aubrey *et al.* 2017 | Access, utilisation and experience of perinatal healthcare | Women preferred care from a female healthcare professional for religious and cultural reasons. Some women would not accept care from male providers and would avoid accessing antenatal care if no female providers available, others would accept care from male providers. | - Huster *et al.* (2014). Cesarean sections among Syrian refugees in Lebanon from December 2012/January 2013 to June 2013: probable causes and recommendations. *The Yale journal of biology and medicine*, *87*(3), 269. - Murray *et al.* (2010). The experiences of African women giving birth in Brisbane, Australia. *Health Care for Women International*, *31*(5), 458-472. - Carroll *et al*. (2007) Caring for Somali women: Implications for clinician–patient communication, *Patient Education and Counseling*, 66(3), 337-345 |
| Balaam *et al.* 2013 | Access, utilisation and experience of perinatal healthcare | Asylum seeker and refugee women’s healthcare experiences were influenced by communication, language and cultural barriers, unfamiliarity with Western health care systems, feelings of stigma and hostility from health professionals, and past experiences. Women had physical and mental health problems, a lack of family and social support, felt vulnerable and had poor socio-economic circumstances. | - Reynolds and White (2010) Seeking Asylum and Motherhood: health and well-being needs. *Community Practitioner* 83(30), 20–23. - Briscoe and Lavender (2009) Exploring Maternity care for asylum seekers and refugees. *British Journal of Midwifery* 17(1), 17–23. - Iliadi (2008) Refugee women in Greece: a qualitative study of their attitudes and experience in antenatal care. *Health Science Journal* 2(3), 173–180. - Kennedy and Murphy-Lawless (2003) The maternity care needs of refugee and asylum seeking women in Ireland. *Feminist Review* 73, 39–53. - Kurth *et al.* (2010) Reproductive health for asylum seeking women – a challenge for health professionals. *BMC Public Health* 10(659), 1–11.) - McLeish (2005) Maternity experiences of asylum seekers in England. *British Journal of Midwifery* 13(12), 782–789. |
| Collins *et al.* 2011 | Perinatal health outcomes (mental health) | Risk factors associated with postnatal depression such as low social support, stressful life events, a previous history of depression and depression during pregnancy, are particularly common among refugees and asylum-seeking women.  The body of literature on this subject needs to be increased, and more consistency is needed in the terms used to differentiate between study populations (e.g. refugees versus asylum-seeking women). Refugee, asylum seeker and immigrant women should be treated as individuals and cannot be regarded as having the same risk of PND as UK-born women, nor as each other. We must avoid the mistake made by much of the literature of treating immigrant women as a homogenous group. | - Stewart *et al.* (2008) Postpartum depression symptoms in newcomers. *The Canadian Journal of Psychiatry*, *53*(2), 121-124. |
| De Maio. 2010. | Perinatal health outcomes (mental health) | Asylum seekers and refugees were significantly more likely than Canadian-born women to achieve scores reflective of higher risk of postpartum depression. Findings indicate a lack of social support, difficulties with language, and/or unfamiliarity with Canadian life and healthcare. | - Stewart *et al.* (2008) Postpartum depression symptoms in newcomers. *The Canadian Journal of Psychiatry*, *53*(2), 121-124. |
| Downe *et al.* 2009 | Access, utilisation and experience of perinatal healthcare | Asylum seekers reported that they did not know how to access care or were under the impression they would have to pay for it. There was a perceived threat to emotional and physical safety experienced by asylum seekers who failed to engage with antenatal services. | - Dartnall *et al.* (2005) Access to Maternity Services Research Report. London: COI and Department of Health. |
| Fellmeth *et al.* 2017 | Perinatal health outcomes (mental health) | Asylum seeker and refugee women experienced higher rates of depression and post-traumatic stress disorder.  Future research should seek to redress the evidence gaps by focusing on refugee and asylum-seeking women. Despite estimates of 59.5 million forcibly displaced persons worldwide, very few studies explicitly focused on refugee and asylum-seeking women. | - Gagnon *et al.* (2013) International migration to Canada: the post-birth health of mothers and infants by immigration class. *Social science & medicine*, *76*, 197-207. - Stewart *et al.* (2008) Postpartum depression symptoms in newcomers. *The Canadian Journal of Psychiatry*, *53*(2), 121-124. |
| Gissler *et al.* 2009 | Perinatal health outcomes (offspring mortality) | The migrant group with high stillbirth, neonatal and infant mortality rates was refugees. Deaths attributed to congenital anomalies, pregnancy complications or intrauterine growth restriction were similarly distributed among refugees and non-refugees, but refugees had a higher preterm rate.  These women represent a high-risk group for obstetrics, calling for special care and attention both during pregnancy and childbirth. The literature suggests several reasons for the poorer outcomes among refugee mothers including more medical problems but fewer interventions than their receiving-country counterparts; a larger burden of poverty-related adverse circumstances; social, communication and emotional problems; different concepts and understanding of health and diseases. All these factors may play a significant role in poorer perinatal and infant outcomes. | - Essén *et al.* (2000) Increased perinatal mortality among sub‐Saharan immigrants in a city‐population in Sweden. *Acta obstetricia et gynecologica Scandinavica*, *79*(9), 737-743. - Kuvacic *et al.* (1996) Possible influence of expatriation on perinatal outcome. *Acta obstetricia et gynecologica Scandinavica*, *75*(4), 367-371. - Lalchandani *et al.* (2001) Obstetric profiles and pregnancy outcomes of immigrant women with refugee status. *Irish Medical Journal*, *94*(3), 79-80. - Schulpen *et al.* (2001) Influences of ethnicity on perinatal and child mortality in the Netherlands. *Archives of disease in childhood*, *84*(3), 222-226. - Vangen *et al.* (2002) Perinatal complications among ethnic Somalis in Norway. *Acta Obstetricia et Gynecologica Scandinavica*, *81*(4), 317-322. - Nedic *et al.* (1999) Deliveries in the outpatient birthing facility in Ruma during 1989 and between 1992 and 1995 [Croatian]. *Medicinski Pregled*. 52:/53-6. |
| Hadgkiss and Renzaho. 2014 | Perinatal health outcomes (offspring mortality, mode of delivery, birthweight, preterm birth, complex obstetric issues) | Asylum seeker women faced a range of complex gynaecological diagnoses and obstetrical issues, including an incidence of severe acute maternal morbidity 4.5 times higher than the general population, were more likely to have experienced sexual assault, had higher rates of unwanted pregnancies and induced abortions than the host population. Longer length of stay was also associated with a lower live birth and abortion rate among asylum seekers. | - Goosen *et al.* (2009) Induced abortions and teenage births among asylum seekers in The Netherlands: analysis of national surveillance data. *Journal of Epidemiology & Community Health*, jech-2008. - Kurth *et al.* (2010) Reproductive health for asylum seeking women – a challenge for health professionals. *BMC Public Health* 10(659), 1–11.) - Van Hanegem *et al.* (2011) Severe acute maternal morbidity in asylum seekers: a two‐year nationwide cohort study in the Netherlands. *Acta obstetricia et gynecologica Scandinavica*, *90*(9), 1010-1016. - Rogstad and Dale (2004) What are the needs of asylum seekers attending an STI clinic and are they significantly different from those of British patients? *Int J STD AIDS*; 15(8): 515–8 |
| Higginbottom *et al.* 2015 | Perinatal health outcomes (mental health); access to and utilisation of perinatal healthcare | Asylum seeker and refugee women faced barriers with care in relation to interpreter services, understanding information given to them, had a lack of knowledge on the availability of support services in the community (e.g. breastfeeding and postpartum depression), felt isolated, lacked adequate social support, lacked economic resources and faced higher rates of postpartum depression. | - Gagnon *et al.* (2007) Refugee and refugee-claimant women and infants post-birth: migration histories as a predictor of Canadian health system response to needs. *Canadian Journal of Public Health/Revue Canadienne de Sante'e Publique*, 287-291. - Merry *et al.* (2011) Refugee claimant women and barriers to health and social services post-birth. *Canadian Journal of Public Health/Revue Canadienne de Sante'e Publique*, 286-290. |
| Higginbottom *et al.* 2012 | Perinatal health outcomes (mental health); access to and utilisation of perinatal healthcare | Refugee women experienced language issues, inconsistent or absence of access to translators, had gender preference for healthcare providers and translators and higher prevalence of postpartum depressive symptoms. | - Redwood-Campbell *et al*. (2008) Understanding the health of refugee women in host countries: lessons from the Kosovar re-settlement in Canada. *Prehospital and Disaster Medicine* 23:322–7. - Stewart *et al*. (2008) Postpartum depression symptoms in newcomers. *Canadian Journal of Psychiatry* 53:121–4. |
| Higginbottom *et al.* 2014 | Access, utilisation and experience of perinatal healthcare | Refugee women’s experiences and satisfaction with maternity healthcare were influenced by having culturally accessible support and procedures, social networks such as families and communities. Healthcare systems are based on a western model of healthcare, some women were confused, dissatisfied with care particularly the role of technology in labour and childbirth and the role and attitude of nurses in the postpartum period. | - Kulig (1990) Childbearing Beliefs Among Cambodian Refugee Women'. *Western Journal of Nursing Research*, *12*(1), 108-118. |
| Mengesha *et al.* 2016 | Access, utilisation and experience of perinatal healthcare | Barriers to accessing care among asylum seeker and refugee women included cultural differences between women and health professionals, limited spoken English, problems in understanding materials written in English, experience with interpreting services, difficulties in accessing information and services, lack information about the healthcare system, difficulty navigating healthcare system, the cost of some services and transportation difficulties. Women also perceived some negatives in their interactions with healthcare providers and perceived racism and/or discrimination within the healthcare system. | - Allotey *et al.* (2004) Reproductive health for resettling refugee and migrant women. *Health issues*, *78*, 12-7. - Carolan and Cassar (2007) Pregnancy care for African refugee women in Australia: attendance at antenatal appointments. *Evidence-Based Midwifery*, *5*(2), 54-59. - Riggs *et al.* (2012) Accessing maternal and child health services in Melbourne, Australia: reflections from refugee families and service providers. *BMC Health Services Research*, *12*(1), 117. - Carolan and Cassar (2010) Antenatal care perceptions of pregnant African women attending maternity services in Melbourne, Australia. *Midwifery* 26.2: 189-201. |
| Merry *et al.* 2013 | Perinatal health outcomes (mode of delivery) | Sub-Saharan African primiparous refugee women had increased risk of having caesareans. An analysis of asylum-seekers who showed no difference in caesarean rates compared to Canadian-born.  More precise definitions of migrants and more complete individual level migration-related data, including source country, length of time in host  country, receiving-country language ability (at the time of pregnancy and birth), and migration status (e.g., refugee or economic immigrant) would allow for better interpretation of results. | - Gagnon *et al.* (2007) Refugee and refugee-claimant women and infants post-birth: migration histories as a predictor of Canadian health system response to needs. *Canadian Journal of Public Health/Revue Canadienne de Sante'e Publique*, 287-291. - Shah *et al*. (2011) Adverse Pregnancy Outcomes Among Foreign-Born Canadians. *J Obstet Gynaecol Can*, 33:207–215 - Helsel *et al.* (1992) Pregnancy among the Hmong: birthweight, age, and parity. *Am J Public Health*, 82:1361–1364 - Richman and Dixon (1985) Comparative study of Cambodian, Hmong, and Caucasian infant and maternal perinatal profiles. *J Nurse* *Midwifery* 30:313–319 - Gann *et al*. (1989) Pregnancy characteristics and outcomes of Cambodian refugees. *Am J Public Health*, 79:1251–1257 |
| Merry *et al.* 2016 | Perinatal health outcomes (mode of delivery) | Mixed evidence regarding whether asylum seeker and refugee women and risk of caesarean: one study reported a reduced risk of emergency caesarean compared with economic and student migrants, and another reported a greater risk of caesarean compared with Canadian-born and other migrant women. | - Gagnon *et al.* (2013) Predictors of emergency cesarean delivery among international migrant women in Canada. *International Journal of Gynecology & Obstetrics*, *121*(3), 270-274. - Kandasamy *et al.* (2014) Obstetric risks and outcomes of refugee women at a single centre in Toronto. *Journal of Obstetrics and Gynaecology Canada*, *36*(4), 296-302. |
| Schmied *et al.* 2017 | Perinatal health outcomes (mental health) | Stresses associated with being a migrant were exacerbated by having a precarious migration status such as being on temporary visas or seeking asylum without legal documentation.  We were surprised little was said about the differences between the experiences of economic migrants and humanitarian migrants. This indicates a significant gap in the literature that requires further work. | - O'Mahony and Donnelly (2013) How does gender influence immigrant and refugee women's postpartum depression help‐seeking experiences? *Journal of psychiatric and mental health nursing*, *20*(8), 714-725. |
| Small *et al.* 2014 | Access, utilisation and experience of perinatal healthcare | Poor communication and inadequate provision of interpretation services led to needs not being met. Inadequate information that could be understood on important topics such as pain management were provided and cultural differences were not accounted for. Experiences of stereotyping and racism from healthcare staff. Healthcare professionals responded inappropriately to traditional female genital cutting demonstrating a lack of knowledge about the issue. | - Bulman and McCourt (2002) Somali refugee women's experiences of maternity care in west London: a case study. *Critical Public Health*, *12*(4), 365-380. - Herrel *et al.* (2004) Somali refugee women speak out about their needs for care during pregnancy and delivery. *Journal of Midwifery & Women’s Health*, *49*(4), 345-349. |
| Tobin *et al.* 2017 | Perinatal health outcomes (mental health) | There is stigma associated with postpartum depression for refugee women. Refugee and immigrant women living described the source of their depressive symptoms to social factors such as family problems or economic hardship rather than biological factors.  The challenge to healthcare providers and policy makers is to raise awareness of postpartum depression in this population. There is a need for continued education of nurses and providers on the unique needs of refugee and immigrant mothers and the multiple ways they may be more susceptible and less able to identify the symptoms and/or seek appropriate help. | - Edge (2006) Perinatal depression: Its absence among Black Caribbean women. *British Journal of Midwifery*, 14, 646-652 - O’Mahony et al. (2012) Barriers and facilitators of social supports for immigrant and refugee women coping with postpartum depression. *Advances in Nursing Science*, 35(3), E42-E56 |
| Villalonga-Olives *et al.* 2016 | Perinatal health outcomes (low birth weight) | No differences between refugees and native born. Surprisingly, there is a lack of studies where the immigrant categories are specified. Hence, we conclude that little is known about what to expect in terms of pregnancy outcomes from asylum seekers.  Several different categories of migrants can be distinguished: asylum-seekers and refugees, victims of trafficking, students, migrant workers, and reunified family members. We are unable to draw any definitive conclusions since only a few studies distinguish between migrant types (e.g. refugees, undocumented migrants). There is a need for studying the health outcomes of this community. | - Lalchandani *et al.* (2001) Obstetric profiles and pregnancy outcomes of immigrant women with refugee status. *Irish Medical Journal*, *94*(3), 79-80. - Small *et al.* (2008) Somali women and their pregnancy outcomes post migration: data from six receiving countries. *BJOG: An International Journal of Obstetrics & Gynaecology*, *115*(13), 1630-1640. |
| Wikberg and Bondas. 2010 | Experience of perinatal healthcare | Cultural differences and racism impacted on asylum seeker and refugee women’s experience with care. Legal status has an important and profuse influence on caring and health, on both a personal and a societal level. It seems that the most vulnerable women with illegal, asylum-seeking, refugee status or from traditionally not accepted minorities or immigration for arranged marriage have the most difficult situation in accessing healthcare and encountering nurses, midwives, and doctors in maternity care. | - Herrel *et al.* (2004) Somali refugee women speak out about their needs for care during pregnancy and delivery. *Journal of Midwifery & Women’s Health*, *49*(4), 345-349. - McLeish (2005) Maternity experiences of asylum seekers in England. *British Journal of Midwifery* 13(12), 782–789. - Nabb (2006). Pregnant asylum-seekers: Perceptions of maternity service provision. *Royal College of Midwives-Evidence-Based Midwifery*, 4(3), 89-95 |
| Winn *et al.* 2017 | Access, utilisation and experience of perinatal healthcare | Barriers to, and experience of, care among asylum seeker and refugee women was influenced by a lack of support from family, friends and husband, information and practical support from health professionals, difficult to navigate healthcare systems, communication barriers, cultural clashes and insensitivity.  There is a lack of literature that specifically focuses on refugee and migrant worker women. Refugees, migrant workers and immigrants are distinct groups, facing unique challenges and barriers to accessing care during pregnancy. | - Herrel *et al.* (2004) Somali refugee women speak out about their needs for care during pregnancy and delivery. *Journal of Midwifery & Women’s Health*, *49*(4), 345-349. - Hill *et al.* (2012) Somali immigrant women’s health care experiences and beliefs regarding pregnancy and birth in the United States. *Journal of Transcultural Nursing*, *23*(1), 72-81. |

**Notes:**

1. Included systematic reviews were omitted from this table if they either did not present any data explicitly for asylum seekers or refuges. The omitted systematic reviews are: Bollini *et al.* 2009; Falah-Hassani *et al.* 2015; Gagnon *et al.* 2009; Nilaweera *et al.* 2014; Pedersen *et al.* 2014; Wittkowski *et al.* 2017; Heaman *et al.* 2013.

2. The references listed represent the included studies in the systematic reviews that were either exclusively on asylum seeker or refugee women, or the systematic review reported data specifically on asylum seekers or refugees from these included studies.
